# The TGF-β ligand DBL-1 is a key player in a multifaceted probiotic protection against MRSA in *C. elegans*

**DOI:** 10.1038/s41598-021-89831-y

**Published:** 2021-05-21

**Authors:** Maria G. M. Mørch, Katrine V. Møller, Marianne O. Hesselager, Rikke H. Harders, Caroline L. Kidmose, Therese Buhl, Kurt Fuursted, Emøke Bendixen, Chong Shen, Lotte G. Christensen, Charlotte H. Poulsen, Anders Olsen

**Affiliations:** 1 Department of Chemistry and Bioscience, Aalborg University, Aalborg, Denmark; 2 Statens Serum Institut, Copenhagen, Denmark; 3 Department of Molecular Biology and Genetics, Aarhus University, Aarhus, Denmark; 4 Gut Immunology Lab, Health & Biosciences , IFF , Brabrand , Denmark

**Keywords:** Biochemistry, Cell biology, Genetics, Microbiology, Systems biology, Molecular medicine

## Abstract

Worldwide the increase in multi-resistant bacteria due to misuse of traditional antibiotics is a growing threat for our health. Finding alternatives to traditional antibiotics is thus timely. Probiotic bacteria have numerous beneficial effects and could offer safer alternatives to traditional antibiotics. Here, we use the nematode *Caenorhabditis elegans* (*C. elegans*) to screen a library of different lactobacilli to identify potential probiotic bacteria and characterize their mechanisms of action. We show that pretreatment with the *Lactobacillus *spp. Lb21 increases lifespan of *C. elegans* and results in resistance towards pathogenic methicillin-resistant *Staphylococcus aureus* (MRSA)*.* Using genetic analysis, we find that Lb21-mediated MRSA resistance is dependent on the DBL-1 ligand of the TGF-β signaling pathway in *C. elegans*. This response is evolutionarily conserved as we find that Lb21 also induces the TGF-β pathway in porcine epithelial cells. We further characterize the host responses in an unbiased proteome analysis and identify 474 proteins regulated in worms fed Lb21 compared to control food. These include fatty acid CoA synthetase ACS-22, aspartic protease ASP-6 and vitellogenin VIT-2 which are important for Lb21-mediated MRSA resistance. Thus, Lb21 exerts its probiotic effect on *C. elegans* in a multifactorial manner. In summary, our study establishes a mechanistic basis for the antimicrobial potential of lactobacilli.

## Introduction

Ever since the discovery of antibiotic substances, microorganisms have shown the ability to develop resistance towards antimicrobial compounds. With the extensive use of antibiotics in agriculture and healthcare systems globally, there is a rapidly growing collection of multidrug-resistant pathogenic bacteria^1^. MRSA is a major cause of nosocomial infections. MRSA is an opportunistic pathogen, often residing on the host without notice. However, infections may originate from preceding asymptomatic colonization or spread to compromised individuals from healthy carriers^2^. The bacteria colonize the surfaces of the host organism such as skin, nasal cavities and the intestine^2,3^. MRSA can result in a versatile spectrum of infections, ranging from superficial to life threatening. As MRSA is resistant to many antibiotics, treating infections has become challenging and will probably lead to increased severe infections and MRSA-related deaths in the future^4^. Therefore, there is an urgent need for alternative treatment strategies.

Probiotic bacteria potentially offer an easy and inexpensive solution as prophylactic treatment to enhance resistance towards MRSA. It is particularly attractive that unlike traditional antibiotics the entire microbiota is not affected by probiotics. The definition of probiotics are living microorganisms that are able to confer a beneficial effect to a host organism when supplemented adequately^5^. Some probiotic mechanisms of action are known: fighting pathogens by competition of anchoring sites^6–8^, signal interference^9^ or secreting antimicrobial agents^10^, maintaining the intestinal barrier integrity^11,12^, and modulation of host immune system^12,13^. It is becoming increasingly clear that the effects of probiotics are strain and species specific and highly diverse^14^. Therefore, to personalize treatment in the future further mechanistic deconvolution of the individual probiotic strains is essential for properly tailored supplementation of strains according to the state of the host organism^15^. The study of individual bacterial strains is often laborious and cost-inefficient in most model systems. However, the small nematode *C. elegans* offers a simple yet powerful way of investigating host-microbe interactions and host responses.

*C. elegans* is a non-parasitic nematode naturally found in rotting fruits and organic matter^16^. The ease of culturing this bacterivore, combined with its short lifecycle and lifespan, its fully annotated genome and available genetic tools have made *C. elegans* a valuable model organism for studying various complex biological processes such as ageing^17^ and innate immunity^18,19^. Being a bacterivore, *C. elegans* can be used to study probiotic effects on a host organism^20^. Lactic acid bacteria have been shown to augment pathogen tolerance in *C. elegans*^21^. Several studies have revealed that the innate immune system of *C. elegans* is often involved in the host response to probiotic bacteria^22–28^. The immune system of *C. elegans* comprises several conserved pathways, one being the evolutionarily conserved TGF-β pathway, which regulates both growth, development and immunity^29^. DBL-1 is one of the ligands of the TGF-β pathway and it regulates growth^30^ and innate immunity^31–33^. DBL-1 signaling controls transcription of several anti-bacterial agents, such as lysosomes and C-lectins^31,34^. Consequently, *dbl-1* deficient mutants are generally more susceptible to a range of pathogens^29^. Recently, DBL-1 signaling was shown to be important for maintaining the natural abundance of members within the *Enterobacter* family in the natural microbiota of *C. elegans*. Interestingly, *dbl-1* deficiency changes the role of these bacteria from commensal to pathogenic^35^.

Here we present the identification of a new probiotic *Lactobacillus *spp*.* Lb21, which both increases longevity and resistance towards MRSA in the *C. elegans* model. Testing factors within the host innate immune pathways revealed that the DBL-1 ligand of the TGF-β pathway was essential to obtain the Lb21-mediated tolerance to MRSA. To broaden our view of potentially important factors, we used mass spectrometry-based protein quantification to assess the changes in protein expression between worms fed Lb21 and worms fed control food. We found that ACS-22, a homologue of human Fatty Acid Transporter 1 (FATP1), VIT-2, a vitellogenin lipoprotein, and ASP-6, an aspartyl protease, were important for the nematodes to obtain full Lb21-mediated protection from MRSA. Taken together, our results suggest a multifaceted mechanism behind the increased pathogenic tolerance elicited by Lb21 preconditioning and a potential pretreatment strategy for MRSA infections.

## Results

### Lb21 extends lifespan in *C. elegans*

**Figure 1:**
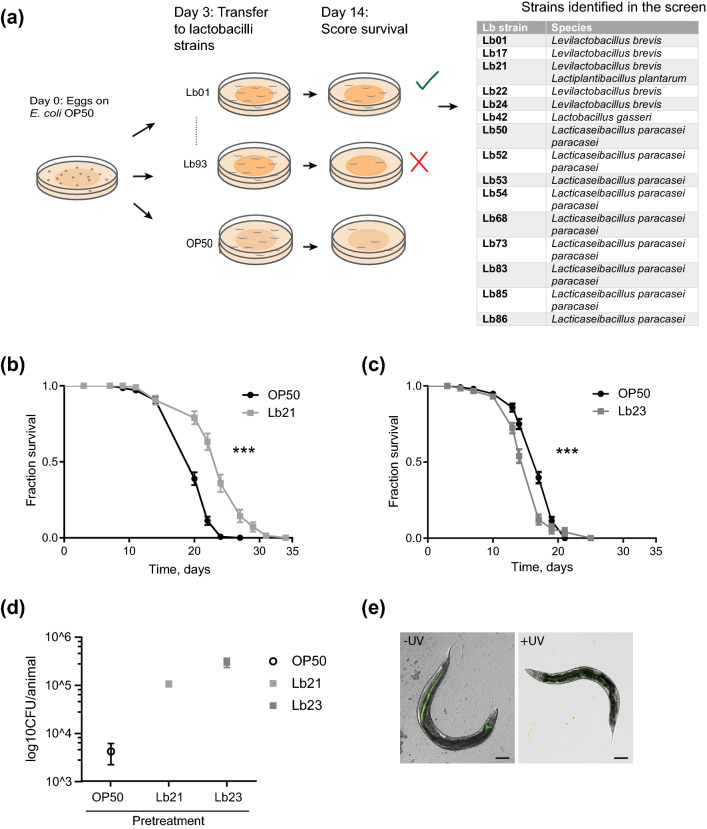
Identifying probiotic lactobacilli strains using *C. elegans*. (**a**) Outline of screening setup to detect bacteria that extend nematode lifespan compared to control bacteria, *E. coli* OP50 and strains isolated in the screen. (**b**) Lifespan of *rrf-3*(*pk1426*) mutants feeding on *Lactobacillus *spp. Lb21 compared to control bacteria, OP50. (**c**) Lifespan of *rrf-3(pk1426)* mutants feeding on *Lactobacillus* strain Lb23 compared to control bacteria, OP50. (**d**) CFU levels on day 5 of animals fed OP50 expressing GFP (OP50-GFP) until adulthood (day 3) and then shifted to Lb21, Lb23 or OP50 for 48 h (n = 10). (**e**) Localization and level of OP50-GFP after 5 days ± UV treatment of plates seeded with OP50-GFP. Scale bar = 100 μm. Graphs show a representative experiment from several independent experiments. See Table S1 for mean ± SD and log-rank test significance.

To find novel probiotic bacteria, we obtained a library of 93 different lactobacilli from DuPont Nutrition Biosciences ApS, and used increased lifespan of *C. elegans* as a screening end-point to limit the number of probiotic candidates (Fig. 1a). To minimize developmental effects the worms were fed the standard food source *Escherichia coli* OP50 until adulthood and subsequently transferred to the individual lactobacilli. After 14 days the plates were scored for populations with increased survival compared to control populations fed OP50. This initial round of screening identified 15 strains with probiotic potential (Fig. 1a). Interestingly, the remaining lactobacilli had no or even detrimental effects on survival so these were not examined further. We decided to focus on one of the beneficial strains, Lb21. Since comparison between two bacterial strains of closer family is likely to confer more information of the underlying mechanisms than comparing more distant families, we included the *Lactobacillus* strain Lb23 for comparison, as it did not increase lifespan. To enable comparison between previously published studies, the standard *C. elegans* feeding strain *E. coli* OP50 was also included as a control strain.

Longitudinal lifespan analyses of Lb21 revealed that mean survival was extended from 9–38% by Lb21 compared to OP50, whereas Lb23 had a 13% decreased mean lifespan compared to OP50 (Fig. 1b,c, Fig S1, and Table S1). Simply shifting worms from an *E. coli* diet to a *Lactobacillus* diet is not per se sufficient to increase lifespan indicating that probiotic effects are highly strain and species specific.

Because Lb21 and Lb23 affected lifespan in opposite directions, we first wanted to establish whether they were both able to colonize *C. elegans* and thereby establish a microbiota in the adult intestine. Using CFU, we evaluated the level of colonization in the intestine of 5-days old adults after 2 days of pretreatment with Lb21, Lb23 or OP50, respectively. In agreement with previous studies^33,36^ we found that OP50 colonized the intestine. Importantly, Lb21 and Lb23 were both able to attach to the intestinal tract and establish a gut microbiota in adult *C. elegans* (Fig. 1d). Hence, the differences in longevity were not a result of one Lactobacillus but not the other being unable to colonize the *C. elegans* intestine.

Establishment of a *C. elegans* microbiota happens during middle age (around day 4) due to declining pharyngeal grinder function^37^. In our pretreatment protocol, we have used OP50 expressing GFP (OP50-GFP) to verify that no OP50 colonization was formed prior to exposure to Lb21 and Lb23. Without UV irradiation of OP50-GFP, the intestinal tract was fully colonized by day 5, whereas no GFP was detected in animals fed UV-treated OP50-GFP (Fig. 1e). Hence, the UV treatment prevented OP50 from colonizing the intestine during development, and the effects of Lb21 and Lb23 are not complicated by intestinal colonization of residual OP50.

To discern the differences between Lb21 and Lb23 we sequenced their genomes (Table S2). This revealed that Lb21 in fact comprised two different *Lactobacillus* species, one *Lactiplantibacillus plantarum* strain (94.3%), referred to as Lb21.1, and one *Levilactobacillus brevis* strain (5.7%), herein referred to as Lb21.2. The Lb23 was confirmed to be a *Levilactobacillus brevis*. Interestingly, a lock-in lifespan analysis showed that Lb21.1 increased longevity compared to Lb21.2, but the mixture of them were superior compared to the individual strains (Fig S2). Therefore, we chose to focus on the Lb21 spp*.* (referred to as Lb21) instead of Lb21.1 and Lb21.2 individually. Some fluctuation in the ratios of the two individual species cannot be avoided, which may explain some of the variation seen in the lifespan analyses (Table S1).

Our screening strategy is based on the general relationship between resistance to different stresses and extended lifespan. Most long-lived animals are also resistant to various stressors^38,39^. Hence, we hypothesized that probiotic bacteria capable of increasing lifespan would likely confer resistance towards pathogenic bacteria such as MRSA. These would be prime candidates for alternatives to traditional antibiotics and thus help reduce the problem of bacterial resistance^1^.

### Lb21 increases MRSA tolerance but not *E. coli* ETEC resistance in vivo

**Figure 2:**
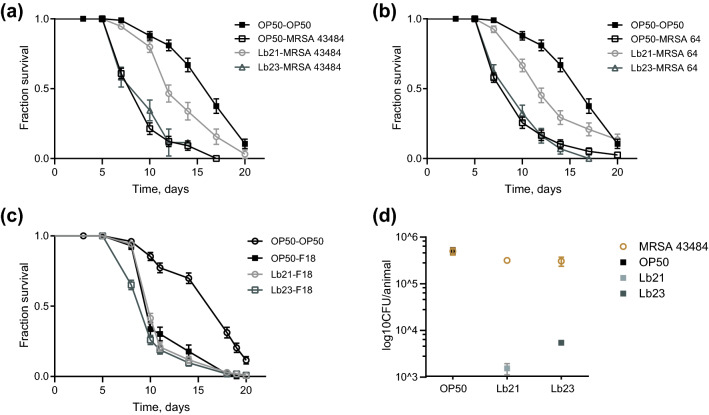
Lb21 but not Lb23 increases MRSA tolerance in *C. elegans*. (**a**) Survival of *rrf-3(pk1426)* mutants pretreated with Lb21, Lb23 and OP50 for 48 h before being shifted to MRSA strain 43484. (**b**) Survival of *rrf-3(pk1426)* mutants pretreated with Lb21, Lb23 and OP50 for 48 h before being shifted to MRSA strain 64. (**c**) Survival of *rrf-3(pk1426) * mutants after pretreatment with Lb21, Lb23 and OP50 for 48 h before being challenged with the pathogen ETEC F18. Graphs show a representative experiment from several independent experiments. See Table S1 for mean ± SD and log-rank test significance. (**d**) CFU level on day 6 of Lb21, Lb23 or OP50 and MRSA 43484 in *rrf-3(pk1426)* mutants pretreated for 48 h with Lb21, LB23 or OP50, respectively (3 technical replicates, n = 10 animals pr. group).

Consistent with our hypothesis, we found that pretreatment with Lb21 for 48 h prior to MRSA challenge significantly enhanced survival of *C. elegans* in an MRSA killing assay compared to control worms. In independent experiments, survival was increased by approximately 40% for two different clinical MRSA isolates, MRSA 43484 and MRSA 64, respectively, compared to OP50-pretreated animals (Fig. 2a,b, Table S1). Despite being a *Lactobacillus* Lb23 provided no or only modestly increased tolerance to MRSA compared to OP50 preconditioning (Fig. 2a,b, and Table S1). Thus, consistent with the longevity result simply shifting the diet from *E.coli* to a *Lactobacillus* does not offer probiotic effects. It needs to be the right *Lactobacillus* strain. In agreement with the observed effect on longevity, the Lb21 spp. provided more resistance to MRSA than the two individual subspecies (Fig S3).

The protective effect of Lb21 on MRSA resistance is remarkable and next we wanted to test the generality of the elicited protection. Many bacteria are pathogenic to *C. elegans* including enterotoxigenic *E. coli* (ETEC). To this end, we pretreated animals with Lb21, Lb23 and OP50 and subsequently exposed them to the gram-negative ETEC F18^40^. Neither Lb21 nor Lb23 were able to protect the nematodes from ETEC F18-induced death (Fig. 2c).

Since the Lb21-mediated protective effect was restricted to MRSA, our next step was to evaluate if Lb21 extended *C. elegans* survival by displacement of MRSA in the intestinal tract. We measured CFU levels in animals exposed to MRSA 43484 for 24 h. Prior to MRSA exposure worms were pretreated with Lb21, Lb23 or OP50 for 48 h. MRSA was equally able to colonize the intestine of *C. elegans* both after Lb21 and Lb23 pretreatment, although residual Lb21 and Lb23 were present at comparable levels (Fig. 2d). Since MRSA can displace the initial colonization by both Lb21 and Lb23 the protecting effect of Lb21 is not a result of merely outcompeting MRSA attachment in the intestinal tract.

Observing a small amount of Lb21 left after MRSA recolonization, we assessed that the positive effect on pathogen resistance could result from bacteria-bacteria interaction rather than from a host-response to Lb21. Therefore, we investigated the growth of MRSA in the presence of Lb21 and Lb23 in vitro (Fig S4). Neither Lb21 nor Lb23 affected the growth of MRSA or vice versa, implying that the decrease in mortality mediated by Lb21 is triggered by a host response and not directly via inter-bacterial interactions. This is consistent with our observation that MRSA colonization is not inhibited by Lb21 pretreatment.

Since Lb21 did not directly impair MRSA colonization the protective effect could be mediated by *C. elegans* responding to a substance secreted by the probiotic bacteria. To test this, we examined the effect of pretreatment with Lb21 bacterial supernatant but it did not confer protection against MRSA (Fig S5 and Table S1). Hence, Lb21 does not secrete an active compound mediating the increased MRSA tolerance. Rather presence of Lb21 cells is needed for at proper host response. Interestingly, UV irradiation of Lb21 did not abolish the protective effect (Fig S5 and Table S1) suggesting that dividing bacteria are not necessary for increased MRSA resistance.

### Lb21 mediates its effect via the DBL-1 ligand of the TGF-β pathway

To better understand the Lb21-mediated host response, we turned to *C. elegans* mutants previously shown to be involved in the responses to probiotic bacteria. We performed MRSA killing assays with mutants of four different anti-aging and immune pathways.

**Figure 3:**
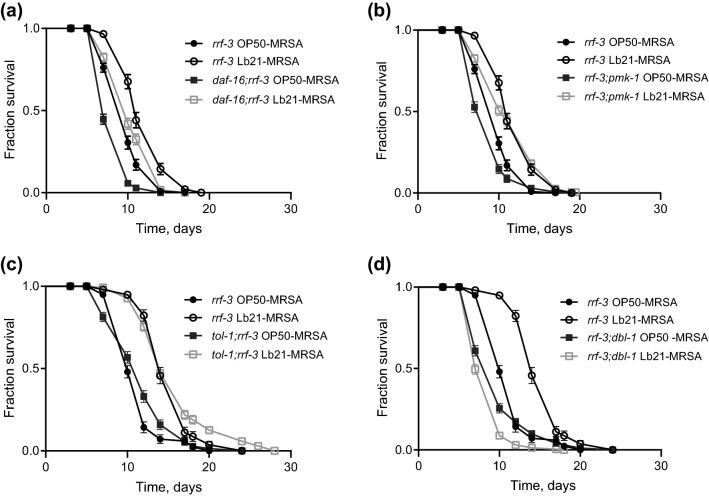
DBL-1 is essential for Lb21-mediated MRSA tolerance. (**a**) Double mutant *daf-16*(*mu86*);*rrf-3*(*pk1426*) survival compared to survival of *rrf-3(pk1426)* pretreated with either OP50 or Lb21 for 48 h before being shifted to MRSA 43484. (**b**) Double mutant *rrf-3*(*pk1426*)*;pmk-1*(*km25*) survival compared to survival of *rrf-3(pk1426)* pretreated with either OP50 or Lb21 before being shifted to MRSA 43484. (**c**) Double mutant *tol-1*(*nr2033*);*rrf-3*(*pk1426*) survival compared to survival of *rrf-3(pk1426)* pretreated with either OP50 or Lb21 before being shifted to MRSA 43484. (**d**) Double mutant *rrf-3*(*pk1426*);*dbl-1*(*nk3*) survival compared to survival of *rrf-3(pk1426)* pretreated with either OP50 or Lb21 before being shifted to MRSA 43484. Graphs show a representative experiment from several independent experiments. See Table S1 for mean ± SD and log-rank test significance.

The forkhead box O (FOXO) transcription factor homologue DAF-16 is negatively regulated by the insulin-like pathway (IIS), and is responsible for transcription of genes involved in extended longevity, innate immunity and stress resistance^41^. We observed that pretreating *daf-16*(*mu86*) mutants with Lb21 increased their tolerance to MRSA compared to OP50-preconditioned control animals (Fig. 3a and Table S1). Thus, Lb21-mediated MRSA tolerance is independent of the DAF-2/DAF-16 IIS. Similarly, neither the p38 MAPK immune pathway nor the toll-like receptor of *C. elegans*, TOL-1, were necessary for the Lb21-mediated response, as Lb21 pretreatment resulted in increased pathogen tolerance in both *pmk-1*(*km25*) and *tol-1*(*nr2033*) mutants (Fig. 3b,c).

Another key immune pathway of *C. elegans* is the TGF-β pathway^29,31^. DBL-1 is one ligand of this pathway and is involved in signaling of both growth and immune responses^29^. Intriguingly, we did not observe an Lb21-induced extension of survival in *dbl-1*(*nk3*) mutants compared to controls (Fig. 3d). Thus, Lb21-mediated MRSA resistance is dependent on DBL-1.

Because probiotic bacteria may exert multifactorial effects, we wondered whether there were other potential modes of actions of Lb21. To identify such mechanisms we decided to examine changes in the proteome following Lb21 pretreatment.

### Unbiased proteome-based discovery of regulated proteins

We used an unbiased approach to investigate the proteomes of Lb21-fed, Lb23-fed and OP50-fed animals by using Mass Spectrometry-based protein identification and relative quantification.

**Figure 4:**
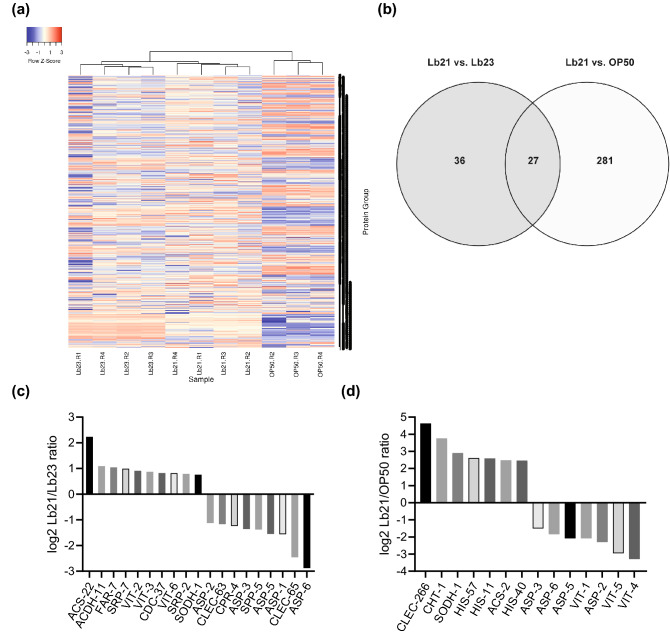
*C. elegans* proteome response to Lb21, Lb23 and OP50. (**a**) Heat map showing hierarchical clustering of the *C. elegans* proteome response to Lb21, Lb23 and OP50, based on log(2) transformed intensities of all quantifiable proteins. (**b**) Venn diagram showing the number of proteins with regulated abundance when two of the three food sources are compared pairwise. A total of 474 proteins showed regulated abundance in either of the comparisons. (**c**) A subset of regulated proteins between Lb21 and Lb23. (**d**) A subset of regulated proteins between Lb21 and OP50. See Table S3 for list of all regulated proteins.

Based on the DDA raw data files and using the ProteinPilot Software (Sciex) we identified 2173 unique *C. elegans* protein groups. These were used to generate a spectral library for protein identifications and relative quantifications across the three sample groups using SWATH data and the Spectronaut (Biognosys) default analysis pipeline. Across all samples, 1980 *C. elegans* proteins (groups) were quantifiable. A heat map representing the hierarchical clustering of the replicates based on log(2) transformed intensities of all proteins illustrates that the samples cluster according to the three groups (Fig. 4a).

In total, 474 proteins showed significant regulation between either of the groups. 308 of the regulated proteins were altered in abundance in Lb21 fed worms versus OP50 fed worms, while only 63 proteins were altered in Lb21 versus Lb23 fed worms (Fig. 4b). Of these, 27 proteins were regulated in both Lb23 and OP50 versus Lb21 (all regulated proteins are listed in Table S3). All regulated proteins were GO annotated and GO enrichment analysis of the 21 proteins upregulated in response to Lb21 compared to Lb23 revealed mainly a biological process enrichment in organic acid metabolic processes and lipid transport and localization. Enrichment analysis of the 42 proteins that were down regulated in Lb21-fed compared to Lb23-fed nematodes, was merely centered on macromolecular catabolic processes and defense response towards other organisms and stimuli (Table 1). Full GO enrichment analysis of all groups in Lb21 compared to OP50 and *P*-values are listed in Table S4.

**Table 1:** GO enrichment analysis between Lb21 and Lb23. Full GO enrichment analysis of all groups in Lb21 compared to OP50 and *P*-values are listed in Table S3.

Up-regulated in Lb21 vs Lb23	Down-regulated in Lb21 vs Lb23
Monocarboxylic acid metabolic process	Proteolysis
Lipid transport	Macromolecule catabolic process
Carboxylic acid metabolic process	Protein catabolic process
Fatty acid metabolic process	Organic substance catabolic process
Oxoacid metabolic process	Organonitrogen compound catabolic process
Lipid localization	Multi-organism process
Organic acid metabolic process	Defense response to other organism
	Response to biotic stimulus
	Response to external biotic stimulus
	Response to other organism
	Defense response
	Catabolic process

### ACS-22, VIT-2 and ASP-6 are involved in Lb21 mediated MRSA resistance

To test the in vivo relevance for MRSA resistance of significantly regulated proteins identified in the proteome analysis, we chose to test the effect of Lb21 pretreatment of mutants for several proteins found to be up or downregulated.

The C-type lectin CLEC-65 and the serpin SRP-7 were found to be down- and upregulated, respectively, in the Lb21/Lb23 proteomic data and the mutants *clec-65*(*ok2337*) and *srp-7*(*ok1090*) were tested for their ability to confer an Lb21 rescuing effect. However, they did not have an effect on Lb21-mediated MRSA tolerance (Table S1).

**Figure 5:**
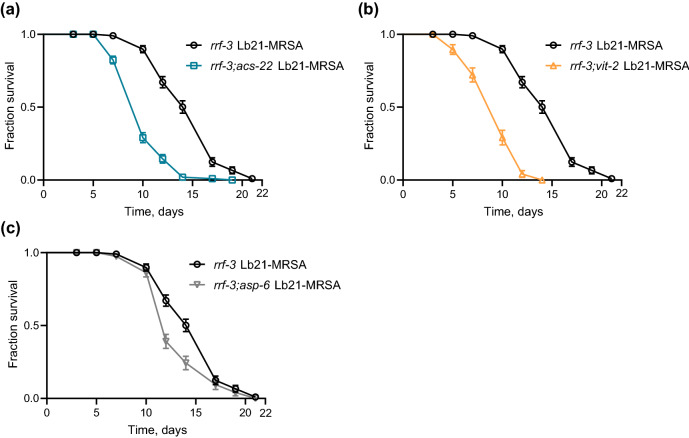
*acs-22*, *vit-2* and *asp-6* are important for the host response to Lb21. (**a**) Double mutant *rrf-3(pk1426); acs-22*(*tm3236*) pretreated with Lb21 before MRSA challenge compared to *rrf-3(pk1426)* pretreated with Lb21 before MRSA challenge. (**b**) Double mutant *rrf-3(pk1426);vit-2*(*ok3211*) pretreated with Lb21 before MRSA challenge compared to *rrf-3(pk1426)* pretreated with Lb21 before MRSA challenge. (**c**) Double mutant *rrf-3(pk1426);asp-6*(*tm2213*) pretreated with Lb21 before MRSA challenge compared to *rrf-3(pk1426)* pretreated with Lb21 before MRSA challenge. See Table S1 for additional data.

The fatty acid CoA synthetase ACS-22 was upregulated in Lb21-fed animals compared to Lb23-fed animals. Therefore, we tested the MRSA resistance of *acs-22*(*tm3236*) mutants pretreated with Lb21, Lb23 and OP50. Intriguingly, Lb21-pretreated *rrf-3*;*acs-22* double mutants did not gain a beneficial effect compared to Lb21-pretreated *rrf-3* controls, rather a decrease in MRSA tolerance ranging from 16–32% was observed in the absence of ACS-22 (Fig. 5a, and Table S1). MRSA tolerance obtained from Lb21 is therefore dependent on ACS-22.

The proteome analysis showed that many vitellogenins were downregulated between Lb21/OP50 and upregulated between Lb21/Lb23 with VIT-2 upregulated between Lb21/Lb23 (Fig. 4c,d, Table S3). Therefore, we evaluated how *vit-2*(*ok3211*) mutants responded to probiotic pretreatment. We found that Lb21 pretreatment of *rrf-3;vit-2* mutants did not result in increased tolerance to MRSA with the mean survival decreased by 35% compared to Lb21 pretreated *rrf-3* controls (Fig. 5b, Table S1). Thus, VIT-2 is part of the Lb21-mediated response, as *vit-2* mutants did not acquire MRSA tolerance.

The aspartic protease ASP-6 was found to be downregulated between both groups in the proteomic analysis (Lb21/OP50 and Lb21/Lb23, Fig. 4c,d). Therefore, we assessed the level of MRSA-induced deaths in Lb21/Lb23/OP50-pretreated *asp-6 *(*tm2213*) mutants. Interestingly, *rrf-3;asp-6* mutants did not obtain full Lb21-mediated MRSA resistance compared to Lb21-pretreated *rrf-3* worms, with the *asp-6* mutants exhibiting a reduction in MRSA tolerance ranging from 12–45% (Fig. 5c, Table S1). This revealed that ASP-6 is required for a part of the Lb21-induced MRSA resistance.

### TGF-β production upon Lb21 administration in an epithelial cell line

There is increasing evidence in mammals suggesting that intestinal epithelial cell-derived TGF-β is regulated by the microbiota^42^. To test if the Lb21-mediated TGF-β response is evolutionarily conserved we examined the response in a cell line to Lb21 administration. As there is a growing problem in the pig production industry of antimicrobial resistance, hereunder MRSA, we chose to use a pig epithelial cell line (IPEC-J2).

**Figure 6:**
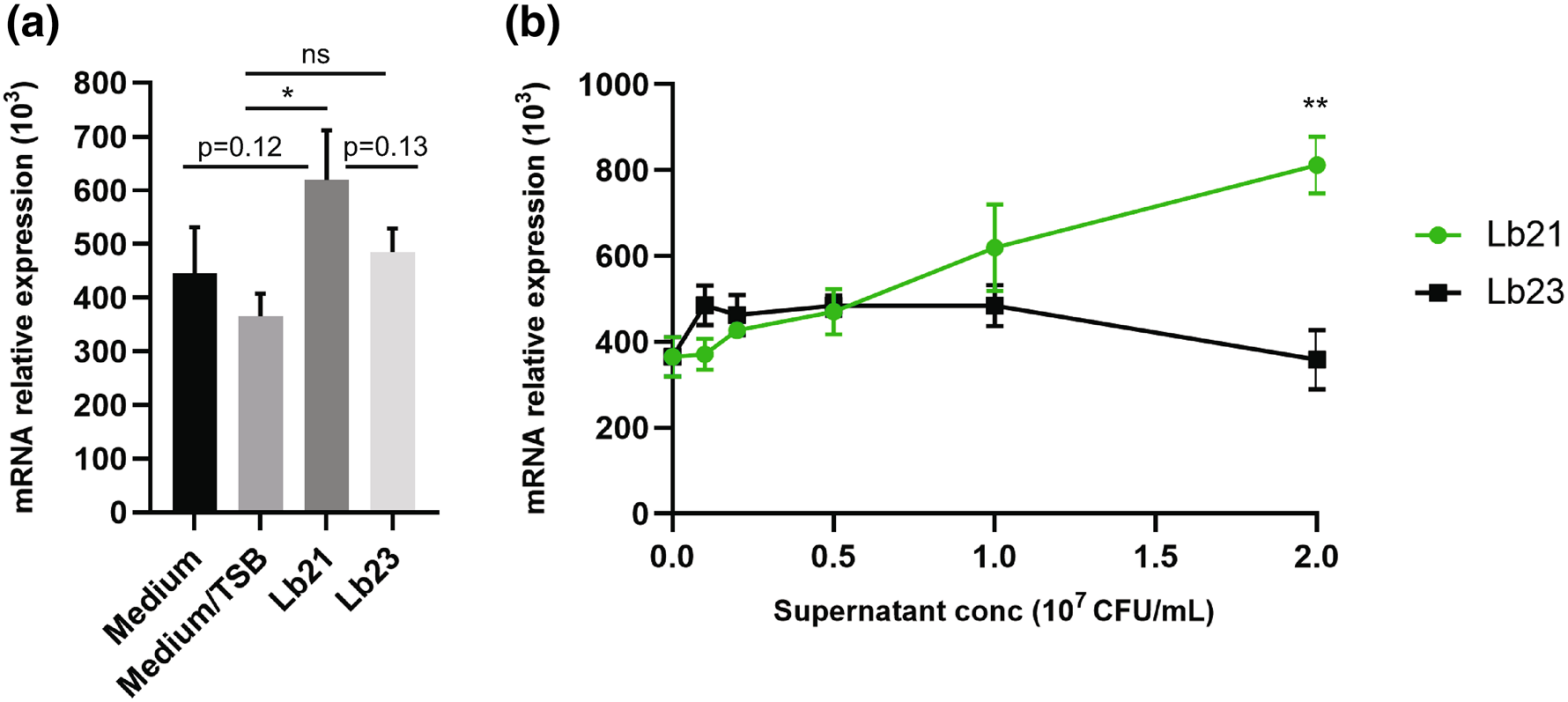
Lb21 but not Lb23 enhanced TGF-β expression of pig epithelial cells (IPEC-J2). Pig epithelial cells (IPEC-J2) were cultured with bacterial supernatant (Lb21 or Lb23, respectively) for 6 h. Cells were then collected and TGF-β mRNA was measured by RT-PCR. The experiments were performed twice with a total of 6 replicates. Data are shown as mean ± SEM. *P < 0.05; **P < 0.01; *n.s.* not statistically significant. (**a**) The TSB media did not increase TGF-β mRNA. However, the Lb21 supernatant at 10^7^ CFU/mL significantly increased TGF-β expression in IPEC-J2 cells. (**b**) TGF-β induction in IPEC-J2 cells by the Lb21 supernatant is dose-dependent.

Using RT-PCR we tested the effect of the bacteria supernatant from Lb21 and Lb23 on IPEC-J2 cells, under different concentrations. The probiotic bacteria were grown in TSB media and initially we verified that this did not increase the expression of TGF-β mRNA (Fig. 6a). At the concentration of 1 × 10^7^ CFU/mL, supernatant of Lb21 but not Lb23 induced a significant higher amount of TGF-β mRNA (*P* < 0.05, Fig. 6a). Interestingly, the supernatant of Lb21 upregulated TGF-β mRNA expression in a dose dependent manner whereas the supernatant of Lb23 did not increase TGF-β mRNA expression (Fig. 6b). At the concentration of 2 × 10^7^ CFU/mL, the supernatant of Lb21 induced a significant higher quantity of TGF-β than that of Lb23 (*P* < 0.01, Fig. 6b).

## Discussion

Probiotics are increasingly attracting attention as dietary health supplements in food and feed but our understanding of their mechanisms of action is incomplete. However, it is clear that among other mechanisms probiotic bacteria can compete with pathogenic bacteria for attachment sites and modulate immune and metabolic responses in the host. It is also evident, that probiotic effects often are strain and species specific, and probiotic bacteria might function as alternatives to traditional antibiotics to combat the increase in multi-resistant bacteria. It is thus relevant to identify novel probiotic bacteria. Here we report identification of a potential probiotic *Lactobacillus *spp. Lb21 as well insight to the mechanisms of action resulting in increased lifespan and MRSA tolerance in *C. elegans.*

**Figure 7:**
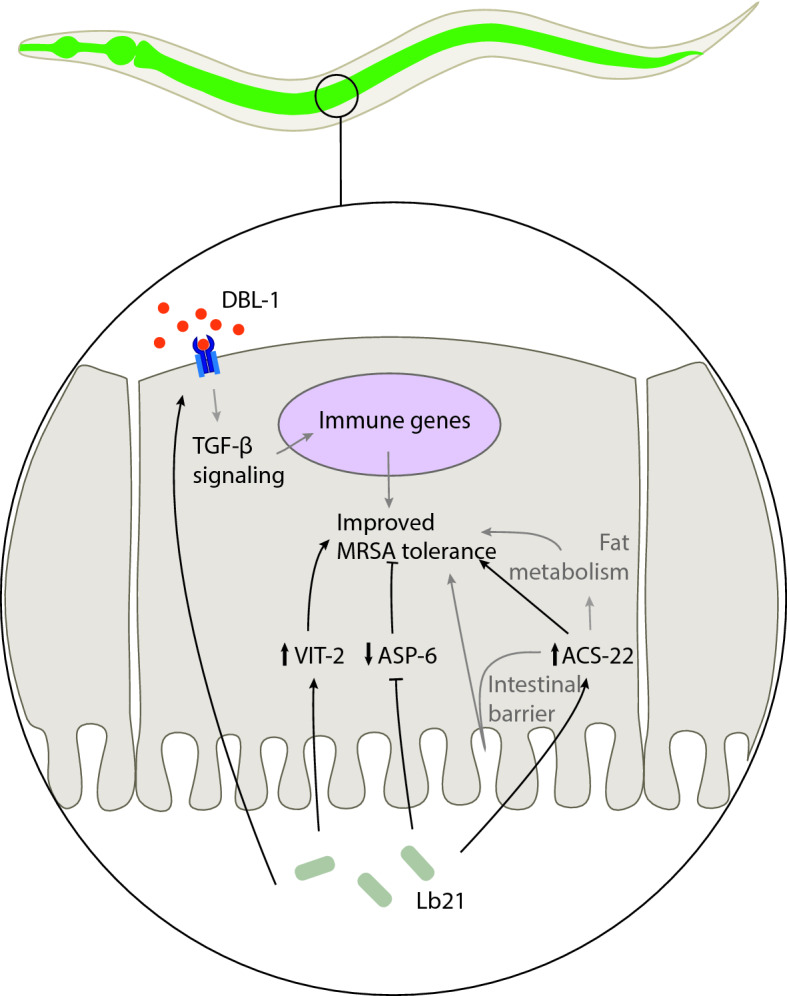
Lb21 affects *C. elegans* in a multifaceted manner. The protection against MRSA mediated by Lb21 is dependent on DBL-1 and the effect could likely be mediated by TGF-β controlled immune genes. MRSA tolerance is also dependent on ACS-22 and the underlying mechanism could be both improved intestinal barrier function and altered fat metabolism. VIT-2 and ASP-6 are also required for the protective effect of Lb21 towards MRSA infection. Black lines indicate interactions shown in this study and grey lines indicate possible mechanisms of action based on published studies.

Combining genetic analysis and unbiased proteomic profiling we find that Lb21-mediated MRSA tolerance is dependent on multiple host factors including DBL-1, the ligand of the TGF-β pathway, ASP-6, VIT-2 and ACS-22. We propose that the Lb21-mediated MRSA resistance is the result of multiple biological processes (Fig. 7).

The involvement of the TGF-β ligand DBL-1 in the probiotic host response is interesting. The TGF-β pathway is a part of the innate immune system and signaling regulates the expression of different immune effectors, such as lectins and lysozymes^29,31^. Our data supports previous studies which have reported that the innate immune system is required for probiotics to have a positive effect^43^. Though DBL-1 is mainly expressed in neurons^30^, the receptors and co-Smads of the TGF-β signaling pathway are highly expressed in the pharynx, epidermis and intestine^29,44^, indicating a role for TGF-β signaling in these tissues. Since the intestinal cells act as the an important line of defense in *C. elegans*^45^, it is possible that Lb21 enhances DBL-1 mediated activation of downstream factors in the intestinal cells and thereby primes the *C. elegans* immune system to resist a subsequent infection. Furthermore, since body size is regulated by DBL-1 TGF-β signaling in the hypodermis^46^, and the fact that DBL-1 signaling regulates anti-microbial peptide expression in the hypodermis^47^, Lb21-modulated DBL-1 activity in the hypodermis could potentially be important for the increased MRSA tolerance possibly through regulation of cuticle collagens^48^. This is also supported by the fact that bacterial colonization of *C. elegans* is regulated via TGF-β signaling in the hypodermis in addition to the pharynx^35^. Additionally, AVA interneurons produce DBL-1 shown to be important for the sensing of bacteria in *C. elegans* and this olfactory learning happens through SMA-6 receptor in the hypodermis^49^. Taken together, DBL-1 signaling could play a role in the recognition of Lb21, if *dbl-1* mutants are unable to sense the bacteria and thus mount a beneficial response.

Involvement of TGF-β signaling in the Lb21 probiotic response was further established when the mRNA expression of TGF-β was only significantly increased in a pig epithelial cell line upon Lb21 supernatant administration and not induced by Lb23. Conservation of probiotic mechanisms between *C. elegans* and pig has previously been reported. A study comparing *C. elegans* and a porcine intestinal epithelial cell line as screening platforms for selecting probiotic bacteria concluded that they were largely able to identify the same probiotic bacteria^50^. Furthermore, similar host responses were induced in both models in agreement with our observations.

In addition to DBL-1, our study identified several other proteins involved in the probiotic response. The *C. elegans* genome comprises six vitellogenin lipoproteins, used in transportation of lipids from the intestine to oocytes to produce yolk^51^. Our proteome analysis identified the vitellogenins VIT-2, -3 and -6 as upregulated between Lb21 and Lb23, whereas VIT-1, -5 and -4 were downregulated between Lb21 and OP50. We confirmed that *vit-2* was necessary to gain full protective effect of Lb21. VIT-2 is thought to be the ancestor of human apolipoprotein B, which is the major component of low density lipoprotein, which also facilitates transport of lipids^52^. Interestingly, there is a possible link between DBL-1 and VIT-2 as TGF-β signaling induces *vit-2* transcription^53^. Consistent with our findings, VIT-2 has also previously been associated with pathogen resistance as *vit-2* deficiency decreases resistance towards the pathogenic bacteria *Photorhabdus luminescens*^54^. It is therefore conceivable that VIT-2 via TGF-β signaling is important in a general immune response. Additionally, morbidity during *C. elegans* ageing is associated with the conversion of gut biomass conversion to yolk^55^. The vitellogenins are generally upregulated between Lb21 and Lb23 but downregulated between Lb21 and OP50, which suggests that moderate level of VITs are more beneficial than high levels of vitellogenins (OP50) or very low levels (Lb23). This is supported by the fact that inhibiting vitellogenesis reduces intestinal atrophy leading to increased lifespan^55^. Furthermore, RNAi inactivation of *vit-2* and *vit-5* increases lifespan^56^. The moderate level of VITs in Lb21-fed nematodes is perhaps advantageous by lowering the intestinal biomass conversion to yolk (intestinal atrophy), while sustaining an immune response via TGF-β.

The lysosome-located proteolytic *asp-6* is primarily expressed in the intestine^57^, and it is upregulated during infections with *Enterococcus faecalis, Erwinia carotovora, P. luminescens* and *Serratia marcescens*^58^*.* We observed that ASP-2, -3, -5 and -6 were downregulated between Lb21/Lb23 and Lb21/OP50 suggesting that Lb21 is not sensed as an infection. Interestingly, *asp-6* deficiency resulted in a negative effect on MRSA tolerance produced by Lb21 compared to control animals likely because APS-6 is needed during the normal response to MRSA infection.

Since ACS-22 was highly upregulated between Lb21 and Lb23, we hypothesized that lack of *acs-22* would abolish the Lb21-mediated MRSA tolerance. Indeed, *acs-22* mutants did not benefit from Lb21 pretreatment. Interestingly, there are several ways that ACS-22 could be required for Lb21-mediated MRSA resistance. ACS-22 is orthologous to the human Fatty Acid Transporter-1/-4 (FATP-1/-4) with predicted long-chain lipid transporter and CoA ligase activity^59^. *acs-22* is primarily expressed in the intestine but also in the pharynx and specific head neurons^59^. Some studies have indicated that ACS-22 has a role in intestinal barrier maintenance^60,61^. Interestingly, a connection between probiotics, intestinal barrier function and *acs-22* was recently reported^61^. Nematodes feeding on the lactic acid bacteria *Lactobacillus bulgaricus* exhibit normal *acs-22* expression in the presence of toxic graphene oxide, whereas control animals have reduced levels of *acs-22*. The authors suggest that the beneficial effect of *L. bulgaricus* is dependent on maintaining a proper intestinal barrier through regulation of ACS-22^61^. Thus, it is plausible that Lb21 exerts its probiotic effects by increasing the integrity of the gut epithelial barrier by upregulating ACS-22. Additionally, ACS-22 has a predicted function in lipid metabolism^59,61,62^ which could also contribute to the probiotic effects of Lb21. The GO enrichment analysis (Table 1) supports this as it showed that processes within lipid transport, metabolism and localization were upregulated in Lb21-fed animals compared to Lb23-fed animals. We also note that several genes within fat metabolism are regulated by DBL-1^63^ and the TGF-β pathway is important for lipid regulation in *C. elegans* as well as in humans and mammals^64,65^. This suggests that there could be a conserved link between ACS-22, VIT-2 and DBL-1 in Lb21-mediated MRSA resistance but the details of such interaction requires further investigation.

With probiotic and microbiota modulation of host health becoming increasingly clear, thorough knowledge and understanding of how the microbes affect their host organism are central for exploitation of probiotics in a correct and meaningful way. Several studies have examined the effect of probiotics alone and in combinations. Some report a detrimental effect of the combinations^66^, whereas others find a synergistic effect of probiotic mixtures^67^. Our sequencing data revealed that Lb21 comprises two different lactobacilli strains from distinct species: 94.3% *L. plantarum* (Lb21.1) and 5.7% *L. brevis* (Lb21.2). Interestingly, the combination of these strains augmented the effect of the individual strains regarding both lifespan extension and MRSA tolerance. We also observed that the genetic background of the host is important for the beneficial effect. This suggest that personalized medicine based approaches in the future could make probiotics even more effective.

## Materials and methods

### Bacterial strains

The lactobacilli strains were provided by DuPont Nutrition Biosciences ApS (Brabrand, Denmark), collected between 1980 and 2014 from various sources, mainly fermented milk, in different countries. The strains used in this study were *Lactobacillus* Lb21 and Lb23. Overnight (ON) cultures of lactobacilli were made by inoculating 75 μL aliquot culture in 10 mL De Man, Rogosa and Sharpe (MRS) broth (SIGMA) at aerobic conditions for 18–24 h depending on the strain under continuous shaking at 250 rpm. Aliquots were made from ON culture prepared from the provided DuPont stocks and stored at -80 °C in 25% glycerol. Nematode growth media (NGM) plates (17 g/L agar (SIGMA), 3 g/L NaCl, 2.5 g/L peptone (BD Difco) with 25 mM KPO_4_ (pH 6.0), 1 mM CaCl_2_, 1 mM MgSO_4_, and 5 μg/mL cholesterol (SIGMA) added after autoclaving) were seeded with 2:1 concentrated ON culture. The *E. coli* OP50 strain was inoculated in 10 mL Luria–Bertani (LB) broth at 37 °C for 18 h under continuous shaking and seeded without concentration. For all bacteria, seeded plates were left to dry for 48 h at room temperature. For supernatant experiments, ON cultures were centrifuged at 3095 rcf for 5 min and the supernatant was pipetted onto dry OP50 plates and left to dry for 48 h at room temperature. Two community-acquired MRSA isolates, MRSA 64 and MRSA 43484 (provided by K. Fuursted, Statens Serum Institut, Copenhagen, Denmark) were spread out from stock aliquot on Tryptic Soy (TS, SIGMA) agar plates and grown ON at 37 °C. Single colonies were inoculated in 10 mL TS broth for 18 h at 37 °C while shaking. The F18 fimbriated enterotoxigenic *E. coli* (ETEC) strain was inoculated in 10 mL LB broth ON at 37 °C while shaking.

### *C. elegans* strains and maintenance

Animals were cultured at 20 °C and maintained on OP50 seeded NGM plates. *C. elegans* strains were purchased from the *Caenorhabditis* Genetic Center (CGC, Minnesota, USA), from the Tokyo Women’s Medical College (Tokyo, Japan) or generated by genetic crossing in our laboratory. *C. elegans* strains used in this study: NL2099 *rrf-3*(*pk1426*)*,* SS104 *glp-4*(*bn2*)*,* OLS77 *daf-16*(*mu86*)*;rrf-3*(*pk1426*), OLS78 *rrf-3*(*pk1426*)*;pmk-1*(*km25*), OLS84 *tol-1*(*nr2033*)*;rrf-3*(*pk1426*)*,* OLS86 *rrf-3*(*pk1426*)*;dbl-1*(*nk3*)*,* OLS114 *rrf-3*(*pk1426*)*;vit-2*(*ok3211*)*,* OLS88 *rrf-3*(*pk1426*)*;asp-6*(*tm2213*)*,* OLS89 *rrf-3*(*pk1426*)*;acs-22*(*tm3236*)*,* OLS117 *rrf-3*(*pk1426*)*;srp-7*(*ok1090*)*,* OLS118 *glp-4*(*bn2*)*;clec-65*(*ok2337*)*. rrf-3* and *glp-4* mutants are sterile at 25 °C and were used to preventing bagging and cross-contamination with offspring. *glp-4* mutants were used instead of *rrf-3* mutants when studying genes on chromosome II and to rule out allele specific effects.

### Genotyping of the *C. elegans* mutants

Prior to experiments, *C. elegans* deletion mutants were verified by PCR. DNA templates were prepared from 1–10 worms incubated for 20 min at − 80 °C in 5 μL of a 100:1 solution of lysis buffer (10 mM Tris pH 8.3, 50 mL KCl, 0.45% NP-40, 0.45% Tween-20, 0.01% gelatin, 2.5 mM MgCl_2_) and proteinase K (10 mg/mL). Worms were lysed in an S1000™ Thermal Cycler (BIORAD) at 60 °C for 45 min and 15 min at 95 °C. DreamTaq MasterMix (FERMENTAS) was used for the PCR. Primers used in this study are shown in Table S5.

### Longitudinal and lock-in lifespan analysis

To synchronize *C. elegans* populations, eggs were harvested from gravid hermaphrodites by means of a hypochlorite solution (0.25 mM KOH, 11.2 mM Sodium Hypochlorite (Sigma)). Worms were washed off NGM plates with S-basal solution (10 M NaCl, 0.05 M KPO_4_ in H_2_O) and centrifuged at 3095 rcf for 1 min, after which the supernatant was removed and 6 mL hypochlorite solution was added, and tubes were shaken vigorously. Eggs were washed in S-basal solution three times before pipetted onto UV-treated OP50 plates.

Synchronized eggs were allowed to develop at 25 °C on UV-treated OP50 for 3 days until adulthood. Hereafter, animals were shifted to the bacteria of interest. For longitudinal lifespan analysis, death events were scored every other day by touch provoked movement and surviving animals were transferred to fresh plates twice a week. For lock-in lifespan analysis, the total number of surviving and dead animals were monitored only once after 14 days.

All lifespan analyses were performed at 25 °C. GraphPad PRISM was used to plot data and perform log-rank test (Gehan-Breslow-Wilcoxon test), and P-values < 0.05 were set as significant. Lost worms were censored.

### Pathogen killing assays

Three-days-old animals were preconditioned with Lb21, Lb23, or OP50 for 48 h before being shifted to MRSA 64, MRSA 43484 or F18 ETEC plates and death events scored every other day. All killing assay were performed at 25 °C. GraphPad PRISM was used to plot data and calculate log-rank test and the Gehan-Breslow-Wilcoxon test, and P-values < 0.05 were set as significant.

### Colony forming units (CFU)

Eggs from hypochlorite treatment of *rrf-3* animals developed into adults on UV-treated GFP expressing OP50 at 25 °C. Three-days-old animals were transferred to OP50, Lb21 and Lb23 for one hour and subsequently moved to fresh plates and left there for 2 days. A subset of the 5-days-old worms were used for colonization measurement and the rest moved to MRSA for 1 day. To see if colonization on MRSA was inhibited by probiotic pretreatment the bacterial load was also determined for 6-day-old worms on MRSA.

To determine colonization levels, ten worms were pooled in an Eppendorf tube with 500 µL S-basal with 0.1% triton X-100, which help disrupt the cuticle of the worms. Samples were made as technical replicates, 10 worms from each of 3 individual plates, collected at once. Worms were allowed to crawl on unspotted NGM plates for 45 min and then washed three times to minimize bacteria associated with the cuticle. A motor pestle was used to release the intestinal bacteria from the worm pellet. The resulting lysates were diluted in S-basal and plated onto selective media according to resistance and incubated overnight at 37 °C. Colonies were quantified and CFU/worm was calculated.

### Proteome analysis

A detailed description can be found in the supporting information. Briefly, 3-days-old *rrf-3* worms, developed on UV-treated OP50, were washed in S-basal and transferred to either Lb21, Lb23 or OP50 seeded NGM plates. On day 5, approximately 1000 worms per replicate were washed in S-basal and gently pelleted. Supernatants were aspirated and worm pellets were snap-frozen in liquid nitrogen and stored at − 80 °C for subsequent protein extraction and proteome analysis. Frozen worm pellets were thawed on ice, lysed, homogenized by beat-beating, and finally soluble protein fractions were recovered. The protein concentration of each sample was adjusted to 1 µg/µL, and enzymatic digestion of the proteins was performed by the addition of trypsin (Sigma-Aldrich/Merck). Proteins were identified and quantified using LC–MS/MS. For each sample, 5 µg peptide material was injected into an Eksigent NanoLC 415 system (AB/Sciex, MA, United States) coupled to TripleTOF 6600 mass spectrometer (AB/Sciex) and a DuoSpray Ion Source (AB/Sciex) controlled by Analyst TF 1.71 software (AB/Sciex).

Replicate analysis (*n* = 4 for Lb21 and Lb23 and *n* = 3 for OP50) of each of the three sample groups was used to generate a spectral library as well as to make protein identifications and relative quantifications.

### Cell line and bacterial supernatant

All cell and bacteria culture media and reagents were purchased from Thermo Fisher Scientific (Roskilde, Denmark), unless otherwise stated. Pig intestinal epithelial cell line (IPEC-J2, ACC 701) was purchased from DSMZ (Braunschweig, Germany). Cells were maintained in DMEM supplemented with 20% FBS, at 37 °C with 5% CO_2_ atmosphere. Cell cultures were supplemented with antibiotics (Penicillin and Streptomycin, 100 ×). The working concentrations in medium was 1 U/mL penicillin and 1 μg/mL of streptomycin.

The bacterial supernatant used in this study were from Lb21 and Lb23. Both bacteria were grown on Tryptic Soy Broth (TSB) at 37 °C under an anaerobic atmosphere (Anaerocult, Merck, Darmstadt, Germany). The supernatant was then collected for cell culture assay. The concentration of the bacterial solution (CFU/mL) was measured by agar plate in parallel.

### Culture conditions and RT-PCR

Pig epithelial cells (IPEC-J2) were cultured with bacterial supernatant (from 0, 0.5… till 2 × 10^7^ CFU/mL) for 6 h and cell pellets were collected for RT-PCR analysis. RNA extraction, quality control and RT-qPCR was performed at Eurofins AROS as described previously^68^. Data are first normalized to two sets of house-keeping genes by following equation: Value = 2^−(Ct sample − Ct house-keeping)^ × 10^3^. The data represent 2 independent experiments with 6 replicates. A student paired t test was used for statistical analysis, assuming two-tail and unequal variance data distribution. The values with statistical significance are indicated. TaqMan assay ID’s are shown in Table S6.

## Supplementary Information

Supplementary Information.

## References

[1] Ventola CL (2015). The antibiotic resistance crisis: Causes and threats. P T J..

[2] Sikorska H, Smoragiewicz W (2013). Role of probiotics in the prevention and treatment of meticillin-resistant *Staphylococcus aureus infections*. Int. J. Antimicrob. Agents.

[3] Vesterlund S, Karp M, Salminen S, Ouwehand AC (2006). *Staphylococcus aureus* adheres to human intestinal mucus but can be displaced by certain lactic acid bacteria. Microbiology.

[4] Stapleton PD, Taylor PW (2007). Europe PMC Funders Group Methicillin resistance in *Staphylococcus aureus*: Methicillin resistance. Sci. Prog..

[5] Araya, M. *et al.* Guidelines for the evaluation of probiotics in food. *Jt. FAO/WHO Work. Gr. Rep. Draft. Guidel. Eval. Probiotics Food* 1–11. 10.1111/j.1469-0691.2012.03873 (2002).

[6] Gueimonde M, Margolles A, de los Reyes-Gavilán CG, Salminen S (2007). Competitive exclusion of enteropathogens from human intestinal mucus by *Bifidobacterium* strains with acquired resistance to bile—A preliminary study. Int. J. Food Microbiol..

[7] Tsai CC (2005). Antagonistic activity against Salmonella infection in vitro and in vivo for two *Lactobacillus* strains from swine and poultry. Int. J. Food Microbiol..

[8] Prince T, McBain AJ, O’Neill CA (2012). *Lactobacillus reuteri* protects epidermal keratinocytes from *Staphylococcus aureus*-induced cell death by competitive exclusion. Appl. Environ. Microbiol..

[9] Piewngam P (2018). Pathogen elimination by probiotic *Bacillus* via signalling interference. Nature.

[10] Zipperer A (2016). Human commensals producing a novel antibiotic impair pathogen colonization. Nature.

[11] O’Flaherty S, Saulnier DM, Pot B, Versalovic J (2010). How can probiotics and prebiotics impact mucosal immunity?. Gut Microbes.

[12] Rhayat L (2019). Effect of *Bacillus**subtilis* strains on intestinal barrier function and inflammatory response. Front. Immunol..

[13] Thomas CM, Versalovic J (2010). Introduction: Probiotic modulation of host signaling pathways. Gut Microbes.

[14] Bermudez-Brito M, Plaza-Díaz J, Muñoz-Quezada S, Gómez-Llorente C, Gil A (2012). Probiotic mechanisms of action. Ann. Nutr. Metab..

[15] Suez J, Zmora N, Segal E, Elinav E (2019). The pros, cons, and many unknowns of probiotics. Nat. Med..

[16] Félix M-A, Braendle C (2010). The natural history of *Caenorhabditis elegans*. Curr. Biol..

[17] Olsen A, Gill MS (2017). Ageing: Lessons from *C. elegans*.

[18] Cohen LB, Troemel ER (2015). Microbial pathogenesis and host defense in the nematode *C. elegans*. Curr. Opin. Microbiol..

[19] Ewbank JJ, Pujol N (2016). Local and long-range activation of innate immunity by infection and damage in *C. elegans*. Curr. Opin. Immunol..

[20] Christensen K, Mørch M, Morthorst T, Lykkemark S, Olsen A, Olsen A, Gill M (2017). Microbiota, probiotic bacteria and ageing. Ageing: Lessons from *C. elegans*.

[21] Ikeda T, Yasui C, Hoshino K, Arikawa K, Nishikawa Y (2007). Influence of lactic acid bacteria on longevity of *Caenorhabditis elegans* and host defense against *Salmonella**enterica* serovar enteritidis. Appl. Environ. Microbiol..

[22] Park MR (2018). Probiotic *Lactobacillus fermentum* strain JDFM216 stimulates the longevity and immune response of *Caenorhabditis elegans* through a nuclear hormone receptor. Sci. Rep..

[23] Zhao L (2017). The transcription factor DAF-16 is essential for increased longevity in *C. elegans* exposed to *Bifidobacterium**longum* BB68. Sci. Rep..

[24] Kamaladevi A, Balamurugan K (2016). *Lactobacillus casei* triggers a TLR mediated RACK-1 dependent p38 MAPK pathway in *Caenorhabditis elegans* to resist *Klebsiella pneumoniae* infection. Food Funct..

[25] Rangan (2016). A bacterial secreted peptidoglycan hydrolase enhances host resistance to intestinal pathogens. Science.

[26] Nakagawa H (2016). Effects and mechanisms of prolongevity induced by *Lactobacillus gasseri* SBT2055 in *Caenorhabditis elegans*. Aging Cell.

[27] Komura T, Ikeda T, Yasui C, Saeki S, Nishikawa Y (2013). Mechanism underlying prolongevity induced by bifidobacteria in *Caenorhabditis elegans*. Biogerontology.

[28] Kim Y, Mylonakis E (2012). *Caenorhabditis elegans* immune conditioning with the probiotic bacterium *Lactobacillus acidophilus* strain ncfm enhances gram-positive immune responses. Infect. Immun..

[29] Gumienny, T. L. & Savage-Dunn, C. TGF-β signaling in *C. elegans*. *Wormb. ed. C. elegans Res. Community, Wormb.* **July**, 1–34 (2013). 10.1895/wormbook.1.22.2 PMC5081272 23908056

[30] Suzuki Y (1999). A BMP homolog acts as a dose-dependent regulator of body size and male tail patterning in *Caenorhabditis elegans*. Development.

[31] Mallo GV (2002). Inducible antibacterial defense system in *C. elegans*. Curr. Biol..

[32] Tenor JL, Aballay A (2008). A conserved Toll-like receptor is required for *Caenorhabditis elegans* innate immunity. EMBO Rep..

[33] Portal-Celhay C, Bradley ER, Blaser MJ (2012). Control of intestinal bacterial proliferation in regulation of lifespan in *Caenorhabditis elegans*. BMC Microbiol..

[34] Mochii M, Yoshida S, Morita K, Kohara Y, Ueno N (1999). Identification of transforming growth factor-β-regulated genes in *Caenorhabditis elegans* by differential hybridization of arrayed cDNAs. Proc. Natl. Acad. Sci. U.S.A..

[35] Berg M (2019). TGFβ/BMP immune signaling affects abundance and function of *C. elegans* gut commensals. Nat. Commun..

[36] Portal-Celhay C, Blaser MJ (2012). Competition and resilience between founder and introduced bacteria in the *Caenorhabditis**elegans* gut. Infect. Immun..

[37] Cabreiro F, Gems D (2013). Worms need microbes too: Microbiota, health and aging in *Caenorhabditis elegans*. EMBO Mol. Med..

[38] Brejning J (2014). Loss of NDG-4 extends lifespan and stress resistance in *Caenorhabditis elegans*. Aging Cell.

[39] Lithgow GJ, White TM, Hinerfeld DA, Johnson TE (1994). Thermotolerance of a long-lived mutant of *Caenorhabditis elegans*. J. Gerontol..

[40] Hesselager MO, Everest-Dass AV, Thaysen-Andersen M, Bendixen E, Packer NH (2016). FUT1 genetic variants impact protein glycosylation of porcine intestinal mucosa. Glycobiology.

[41] Tullet JMA (2015). DAF-16 target identification in *C. elegans*: Past, present and future. Biogerontology.

[42] Bauché D, Marie JC (2017). Transforming growth factor β: A master regulator of the gut microbiota and immune cell interactions. Clin. Transl. Immunol..

[43] Kwon G, Lee J, Koh JH, Lim YH (2017). Lifespan extension of *Caenorhabditis elegans* by *Butyricicoccus pullicaecorum* and *Megasphaera elsdenii* with probiotic potential. Curr. Microbiol..

[44] Roberts AF, Gumienny TL, Gleason RJ, Wang H, Padgett RW (2010). Regulation of genes affecting body size and innate immunity by the DBL-1/BMP-like pathway in *Caenorhabditis elegans*. BMC Dev. Biol..

[45] Pukkila-Worley R, Ausubel FM (2012). Immune defense mechanisms in the *Caenorhabditis elegans* intestinal epithelium. Curr. Opin. Immunol..

[46] Schultz RD, Bennett EE, Ellis EA, Gumienny TL (2014). Regulation of extracellular matrix organization by BMP signaling in *Caenorhabditis elegans*. PLoS ONE.

[47] Zugasti O, Ewbank JJ (2009). Neuroimmune regulation of antimicrobial peptide expression by a noncanonical TGF-β signaling pathway in *Caenorhabditis elegans* epidermis. Nat. Immunol..

[48] Madaan U (2018). BMP signaling determines body size via transcriptional regulation of collagen genes in *Caenorhabditis elegans*. Genetics.

[49] Zhang X, Zhang Y (2012). DBL-1, a TGF-, is essential for Caenorhabditis elegans aversive olfactory learning. Proc. Natl. Acad. Sci..

[50] Zhou M (2014). Investigation into in vitro and in vivo models using intestinal epithelial IPEC-J2 cells and *Caenorhabditis elegans* for selecting probiotic candidates to control porcine enterotoxigenic *Escherichia**coli*. J. Appl. Microbiol..

[51] Perez MF, Lehner B (2019). Vitellogenins—Yolk gene function and regulation in *Caenorhabditis elegans*. Front. Physiol..

[52] Baker ME (1988). Is vitellogenin an ancestor of apolipoprotein B-100 of human low-density lipoprotein and human lipoprotein lipase?. Biochem. J..

[53] Goszczynski B, Captan VV, Danielson AM, Lancaster BR, McGhee JD (2016). A 44 bp intestine-specific hermaphrodite-specific enhancer from the *C. elegans**vit-2* vitellogenin gene is directly regulated by ELT-2, MAB-3, FKH-9 and DAF-16 and indirectly regulated by the germline, by *daf-2*/insulin signaling and by the TGF-β/Sma/Mab pa. Dev. Biol..

[54] Fischer M, Regitz C, Kull R, Boll M, Wenzel U (2013). Vitellogenins increase stress resistance of *Caenorhabditis elegans* after *Photorhabdus**luminescens* infection depending on the steroid-signaling pathway. Microbes Infect..

[55] Ezcurra M (2018). *C. elegans* eats its own intestine to make yolk leading to multiple senescent pathologies. Curr. Biol..

[56] Murphy CT (2003). Genes that act downstream of DAF-16 to influence the lifespan of *Caenorhabditis elegans*. Nature.

[57] Lochnit G, Grabitzki J, Henkel B, Tavernarakis N, Geyer R (2006). First identification of a phosphorylcholine-substituted protein from *Caenorhabditis elegans*: Isolation and characterization of the aspartyl protease ASP-6. Biol. Chem..

[58] Wong D, Bazopoulou D, Pujol N, Tavernarakis N, Ewbank JJ (2007). Genome-wide investigation reveals pathogen-specific and shared signatures in the response of *Caenorhabditis elegans* to infection. Genome Biol..

[59] Kage-Nakadai E (2010). Two very long chain fatty acid acyl-CoA synthetase genes, acs-20 and acs-22, have roles in the cuticle surface barrier in *Caenorhabditis elegans*. PLoS ONE.

[60] Qu M, Xu K, Li Y, Wong G, Wang D (2018). Using *acs-22* mutant *Caenorhabditis elegans* to detect the toxicity of nanopolystyrene particles. Sci. Total Environ..

[61] Zhao Y (2015). Lactic acid bacteria protects caenorhabditis elegans from toxicity of graphene oxide by maintaining normal intestinal permeability under different genetic backgrounds. Sci. Rep..

[62] Rourke EJO, Soukas AA, Carr CE (2010). *C. elegans* major fats are stored in vesicles distinct from lysosome-related organelles. Cell.

[63] Liang J, Yu L, Yin J, Savage-Dunn C (2007). Transcriptional repressor and activator activities of SMA-9 contribute differentially to BMP-related signaling outputs. Dev. Biol..

[64] Clark JF, Meade M, Ranepura G, Hall DH, Savage-Dunn C (2018). *Caenorhabditis elegans* DBL-1/BMP regulates lipid accumulation via interaction with insulin signaling. G3 Genes Genomes Genet..

[65] Böttcher Y (2009). Adipose tissue expression and genetic variants of the bone morphogenetic protein receptor 1A gene (BMPR1A) are associated with human obesity. Diabetes.

[66] Collado MC, Grześkowiak Ł, Salminen S (2007). Probiotic strains and their combination inhibit in vitro adhesion of pathogens to pig intestinal mucosa. Curr. Microbiol..

[67] Sniffen JC, McFarland LV, Evans CT, Goldstein EJC (2018). Choosing an appropriate probiotic product for your patient: An evidence-based practical guide. PLoS ONE.

[68] Shen C, Christensen LG, Bak SY, Christensen N, Kragh K (2020). Immunomodulatory effects of thymol and cinnamaldehyde in chicken cell lines. J. Appl. Anim. Nutr..

