# Supplementary material for: The TGF-β ligand DBL-1 is a key player in a multifaceted probiotic protection against MRSA in C. elegans
Source: Sci Rep. 2021 May 21;11:10717. doi: 10.1038/s41598-021-89831-y (PMC8139972; doi:10.1038/s41598-021-89831-y)
Supplement: Supplementary file 1 — Supplementary Information. [file 41598_2021_89831_MOESM1_ESM.pdf]

Supporting information for

The TGF-β ligand DBL-1 is a key player in a multifaceted probiotic protection against MRSA in *C. elegans*

Maria G. M. Mørch[1+], Katrine V. Møller[1+], Marianne O. Hesselager[1+], Rikke H. Harders[1], Caroline L. Kidmose[1], Therese Buhl[1], Kurt Fuursted[2], Emøke Bendixen[3], Chong Shen[4], Lotte G. Christensen[4], Charlotte H. Poulsen[4], Anders Olsen[1*]

[1] Department of Chemistry and Bioscience, Aalborg University, Aalborg, Denmark.
[2] Statens Serum Institut, Copenhagen, Denmark.
[3] Department of Molecular Biology and Genetics, Aarhus University, Aarhus, Denmark.
[4] Gut Immunology Lab, Technology & Innovation, DuPont Nutrition & Biosciences, Aarhus, Denmark.

+ These authors contributed equally to this work.

Keywords: *Caenorhabditis elegans*, probiotic bacteria, *Lactobacillus*, TGF-β, MRSA, proteomics.

* Corresponding Author:

E-mail: ao@bio.aau.dk

Supplementary figures:

Figure S1. Combined lifespan data from six independent experiments. All lifespan data from *rrf-3* mutants fed OP50 and Lb21 analyzed in one data set. For OP50 n= 823, and for Lb21 n = 648.

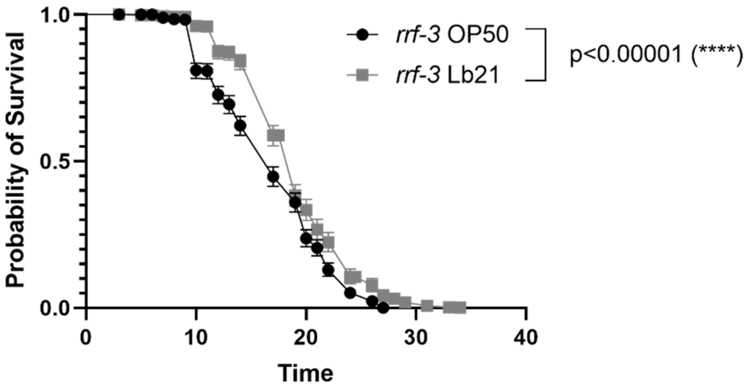

Figure S2. Longevity analysis of Lb21, Lb21.1 (*L. plantarum*), and Lb21.2 (*L. brevis*).
The individual strains of Lb21 are not able to extend the lifespan of *C. elegans* to the same level as the combination. Animals alive at day 18 in each group OP50: 25/88, LB21: 55/99, LB21.1: 42/117, and LB21.2: 8/73.

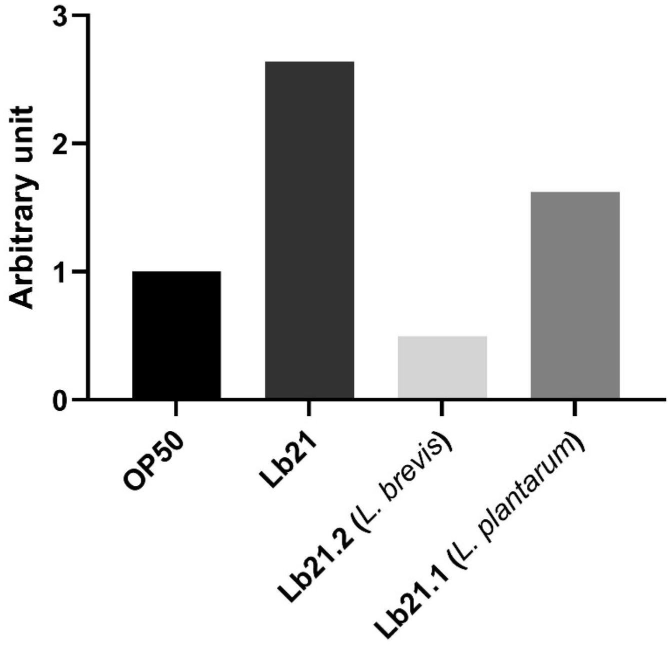

Figure S3. MRSA protection with Lb21 individual strains.
The individual strains of Lb21 are not able to protect *C. elegans* against MRSA infection to the same degree as the combination. Lb21.1 *(L. plantarum),* Lb21.2 *(L. brevis).* To obtain sterile strains the *rrf-3* (**a**) and *glp-4* (**b**) mutants were used.

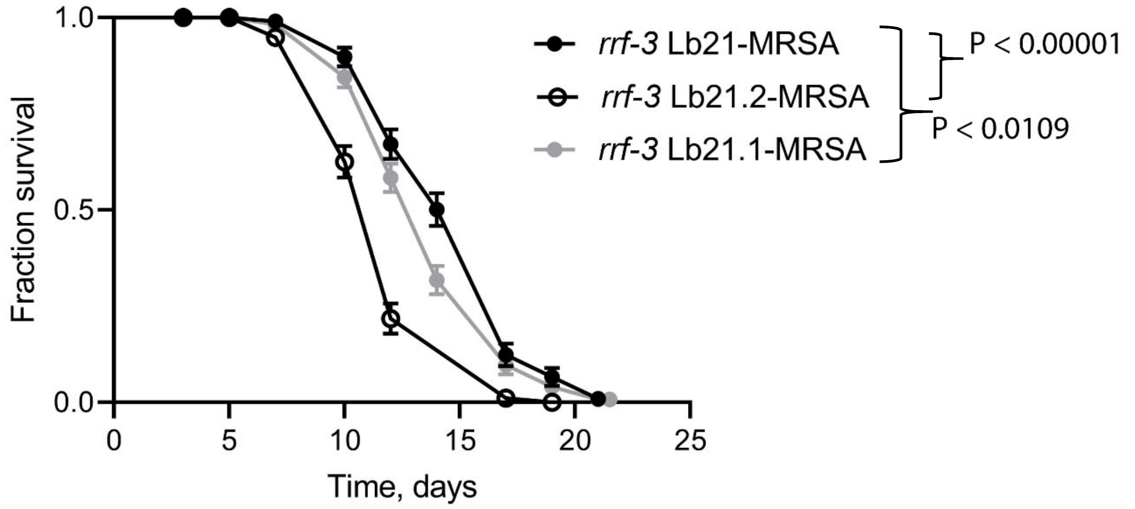

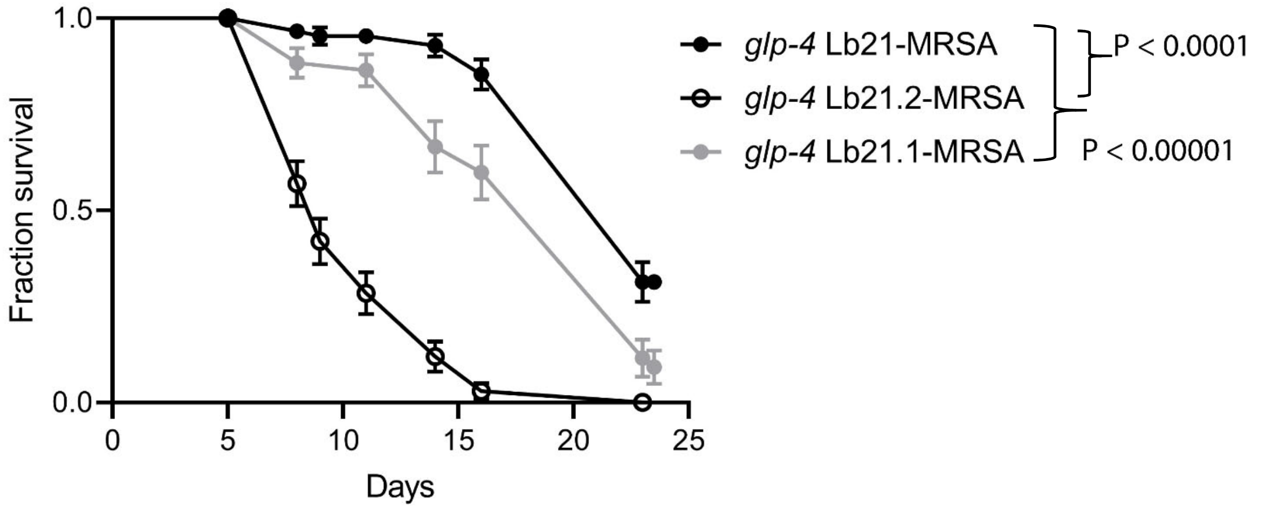

Figure S4. Lb21 and Lb23 does not inhibit pathogenic bacterial growth *in vitro*.
The effect of Lb21 and Lb23 on MRSA 43484 and F18 ETEC growth was assessed at 25˚C. No inhibitory effect was observed.

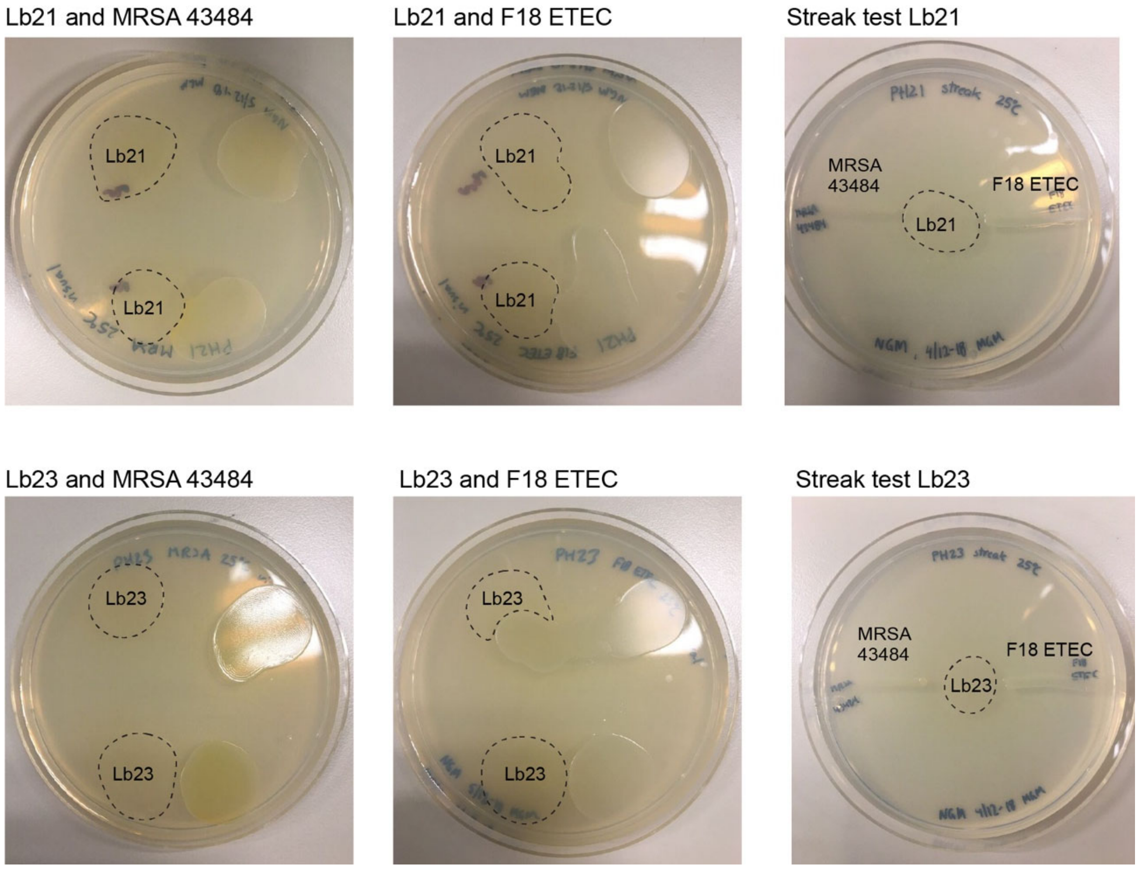

Figure S5. Lb21 does not secrete an antimicrobial agent.
Spend media (Supernatant – Lb21Sup) did not provide protection against MRSA. UV irradiation of Lb21 did not remove the protective effect.

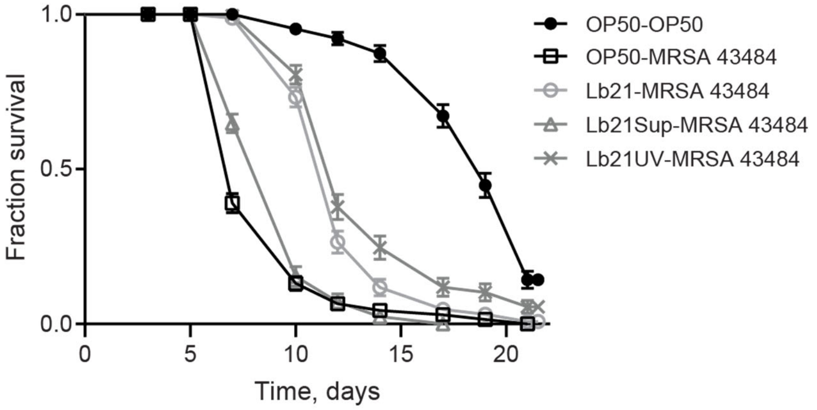

Supplementary tables:

Table S1. All lifespans and MRSA assays (Mean survival, p-value and no. of animals).

Strain	Bacteria	Mean survival Days ±SD	P-Value Log-rank test compared to OP50 or OP50-MRSA	P-value Log-rank test between specified groups	No. of animals	Experiment no.
rrf-3	OP50	20.3 ± 2.9			134	1
	Lb21	23.4 ± 4.8	<0.0001 (****)		74	
rrf-3	OP50	14.1 ± 4.9			77	2
	Lb21	19.5 ± 5.2	<0.0001(***)		86	
rrf-3	OP50	16.6 ± 5.5			193	3
	Lb21	19.7 ± 3.7	<0.0001 (***)		132	
rrf-3	OP50	13.6 ± 3.1			77	4
	Lb21	15.0 ± 2.8	<0.0001(***)		161	
rrf-3	OP50	15.5 ± 4.9			114	5
	Lb21	17.1 ± 4.5	ns (0.1207)		21	
rrf-3	OP50	16.4 ± 4.0			228	6
	Lb21	17.9 ± 3.2	0.0002 (***)		174	
rrf-3	OP50	16.7 ± 3.1			170	7
	Lb23	14.5 ± 3.4	<0.0001 (***)		112	
rrf-3	OP-MRSA 64	7.5 ± 1.6			191	8
	Lb21-MRSA 64	10.3 ± 2.7	<0.0001 (***)	Lb21 vs Lb23: <0.0001 (****)	123	
	Lb23-MRSA 64	8.3 ± 2.1		Lb23 vs OP50: 0.0102 (*)	202	
	OP-MRSA 64	8.7 ± 2.7	<0.0001 (****)		110	9
rrf-3	Lb21-MRSA 64	11.9 ± 3.3		Lb21 vs Lb23: <0.0001 (****)	59	
	Lb23-MRSA 64	9.2 ± 2.6		Lb23 vs OP50: ns	31	
rrf-3	OP-MRSA 43484	8.5 ± 2.1	<0.0001 (****)		110	10
	Lb21-MRSA 43484	12.2 ± 3.5		Lb21 vs Lb23: <0.0001 (****)	59	
	Lb23-MRSA 43484	8.2 ± 1.7		Lb23 vs OP50: ns	31	
	OP-MRSA 43484	8.3 ± 2.4			226	11
rrf-3	Lb21-MRSA 43484	12.1 ± 2.4	<0.0001(****)	Lb21 vs Lb21sup: <0.0001 (****)	154	
	Lb21Sup-MRSA 43484	8.7 ± 2.1	0.0001 (***)	LB21UV vs LB21Sup: 0.0001 (****)	160	
	Lb21UV-MRSA 43484	12.7 ± 2.8	<0.0001 (****)	Lb21UV vs Lb21: 0.0018 (**)	132	
rrf-3	OP-MRSA 43484	9.5 ± 2.3	<0.0001 (****)		153	12
	Lb21-MRSA 43484	12.2 ± 2.8			110	
daf-16;rrf-3	OP-MRSA 43484	8.0 ± 1.6	<0.0001 (****)		205	
	Lb21-MRSA 43484	10.7 ± 2.6			202	
rrf-3;pmk-1	OP-MRSA 43484	8.5 ± 2.3	<0.0001 (****)		190	
	Lb21-MRSA 43484	11.5 ± 3.3			234	

Strain	Bacteria	Mean survival Days ±SD	P-Value Log-rank test compared to OP50 or OP50-MRSA	P-value Log-rank test between specified groups	No. of animals	Experiment no.
rrf-3	OP-MRSA 43484	7.5 ± 1.1			179	13
	Lb21-MRSA 43484	13.4 ± 3.2	<0.0001 (****)	Lb21 vs Lb23: 0.0117 (*)	152	
	Lb23-MRSA 43484	12.6 ± 3.1	<0.0001 (****)		50	
daf-16;rrf-3	OP-MRSA 43484	8.4 ± 1.9			64	
	Lb21-MRSA 43484	12.1 ± 3.0	<0.0001 (****)	Lb21 vs Lb23: 0.0001 (****)	153	
	Lb23-MRSA 43484	8.2 ± 1.9	ns (0.5297)		45	
rrf-3;pmk-1	OP-MRSA 43484	7.8 ±1.4			161	
	Lb21-MRSA 43484	10.8 ± 2.9	<0.0001 (****)	Lb21 vs Lb23: 0.0001 (****)	165	
	Lb23-MRSA 43484	8.5 ± 2.8	0.0018 (**)		76	
rrf-3	OP-MRSA 43484	10.8 ± 2.2			138	14
	Lb21-MRSA 43484	14.8 ± 3.1	<0.0001 (****)		99	
tol-1;rrf-3	OP-MRSA 43484	11.5 ± 3.5			172	
	Lb21-MRSA 43484	15.8 ± 4.5	<0.0001 (****)		214	
rrf-3;dbl-1	OP-MRSA 43484	9.8 ± 3.3	<0.0001 (****)		222	
	Lb21-MRSA 43484	8.7 ± 2.0			260	
rrf-3	OP-MRSA 43484	9.4 ± 1.8			167	15
	Lb21-MRSA 43484	10.1 ± 1.4	<0.0001 (****)		58	
tol-1;rrf-3	OP-MRSA 43484	9.9 ± 2.1			129	
	Lb21-MRSA 43484	11.2 ± 1.8	<0.0001 (****)		79	
rrf-3;dbl-1	OP-MRSA 43484	8.0 ± 1.6	0.0037 (**)		234	
	Lb21-MRSA 43484	7.6 ± 1.2			227	
rrf-3	OP-MRSA 43484	10.9 ± 2.9			116	16
	Lb21-MRSA 43484	20.0 ± 7.8	<0.0001 (****)		47	
rrf-3;asp-6	OP-MRSA 43484	9.9 ± 2.7			93	
	Lb21-MRSA 43484	11.0 ± 2.5	<0.0001 (****)		60	
rrf-3	OP-OP	12.9 ± 3.1			190	17
	OP-MRSA 43484	7.6 ± 1.3			195	
	Lb21-MRSA 43484	14.6 ± 3.1	<0.0001 (****)	Lb21 vs Lb23: <0.0001 (****)	128	
	Lb23-MRSA 43484	10.3 ± 2.3	<0.0001 (****)		158	
rrf-3;asp-6	OP-OP	12.6 ± 2.9			227	
	OP-MRSA 43484	8.5 ± 2.1			202	
	Lb21-MRSA 43484	12.8 ± 3.1	<0.0001 (****)	Lb21 vs Lb23: <0.0001 (****)	96	
	Lb23-MRSA 43484	9.9 ± 2.6	<0.0001 (****)		122	
rrf-3;acs-22	OP-OP	8.3 ± 1.8			164	
	OP-MRSA 43484	8.0 ± 1.4			180	
	Lb21-MRSA 43484	10.0 ± 2.1	<0.0001 (****)	Lb21 vs. Lb23: 0.4356 (ns)	164	
	Lb23-MRSA 43484	9.8 ± 2.4	<0.0001 (****)		99	
rrf-3;vit-2	OP-OP	9.3 ± 2.2			92	
	OP-MRSA 43484	8.3 ± 2.1			106	
	Lb21-MRSA 43484	9.5 ± 2.4	<0.0001 (****)	Lb21 vs. Lb23: <0.0001 (****)	79	
	Lb23-MRSA 43484	7.3 ± 1.9	0.0029 (**)		53	

Strain	Bacteria	Mean survival Days ±SD	P-Value Log-rank test compared to OP50 or OP50-MRSA	P-value Log-rank test between specified groups	No. of animals	Experiment no.
rrf-3	OP-OP	16.5 ±2.6			56	18
	OP-MRSA 43484	8.3 ± 2.2			215	
	Lb21-MRSA 43484	13.9 ±3.3	<0.0001(****)		43	
rrf-3;acs-22	OP-OP	17.4 ± 3.3			73	
	OP-MRSA 43484	8.8 ± 2.2			195	
	Lb21-MRSA 43484	11.7 ± 3.4	<0.0001(****)	*rrf-3* Lb21-MRSA vs *acs-22* Lb21-MRSA: 0.0067 (**)	174	
rrf-3;srp-7	OP-OP	16.7 ± 3.0			79	
	OP-MRSA 43484	10.0 ± 2.8			179	
	Lb21-MRSA 43484	13.4 ± 4.4	<0.0001 (****)	*rrf-3* Lb21-MRSA vs *rrf-3;srp-7* Lb21-MRSA: ns	80	
rrf-3	OP-OP	12.9 ± 3.1			190	19
	OP-MRSA 43484	7.6 ± 1.3			195	
	Lb21 *L. brevis*-MRSA 43484	11.7 ± 2.8		Lb21 vs Lb21 *L. plantarum*: <0.0109 (*)	115	
	Lb21 *L. plantarum*-MRSA 43484	13.6 ± 3.0		Lb21 *L.brevis* vs Lb21 *L. plantarum*: <0.0001 (****)	160	
	Lb21-MRSA 43484	14.6 ± 3.1		Lb21 vs Lb21 *L. brevis*: <0.0001 (****)	128	
glp-4	OP-OP	19.7 ± 5.1			107	20
	OP-MRSA 43484	11.1 ± 3.7			128	
	Lb21 *L. brevis*-MRSA 43484	10.6 ± 3.5		Lb21 vs Lb21 *L. brevis*: <0.0001 (****)	69	
	Lb21 *L. plantarum*-MRSA 43484	17.6 ± 6.0		Lb21 *L. brevis* vs Lb21 *L. plantarum*: <0.0001 (****)	43	
	Lb21-MRSA 43484	20.8 ± 4.4		Lb21 vs Lb21 *L. plantarum*: <0.0001 (****)	55	
glp-4;clec-65	OP-OP	17.8 ± 4.2			123	21
	OP-MRSA 43484	10.8 ± 2.4			92	
	Lb21-MRSA 43484	21.2 ± 4.1	<0.0001 (****)	*glp-4* Lb21-MRSA vs *glp-4;clec-65* Lb21-MRSA: ns	68	
	Lb23-MRSA 43484	13.8 ± 5.9	<0.0001 (****)		77	

Table S2. Basic statistics of the genome assembly of Lb21 and Lb23.

Genome	Classification by GTDB	Genome size	GC content (%)	Longest scaffold	Scaffold N50	# Contigs	# Ns	Completeness (%)	Contamination (%)
Lb21 (CP463_03)	*L plantarum*	5350930	45.3	275932	49970	215	0	100.00	100.00
Lb21.1 (CP463_03_subset1)	*L plantarum*	2949087	44.8	275932	84333	76	0	99.07	2.78
Lb21.2 (CP463_03_subset2)	*L brevis*	2431927	45.9	80868	30857	139	0	98.44	0.00
Lb23 (CP463_04)	*L brevis*	2478959	45.8	470263	329543	26	0	99.06	0.00

Genome size is the total size of all scaffolds (bp). **GC content** is the percentage of the genome consisting of GC. **Longest scaffold** is the longest scaffold in the assembly (bp). **Scaffold N50** is a median statistic that indicates that 50% of the entire assembly is included in scaffolds equal to or larger than this size (bp). **# Scaffolds** is the number of scaffolds the assembly consists of. **# Contigs** is the number of contigs the assembly consists of. **# Ns** is the number of ambiguous bases (N) in the assembly. **Completeness** is estimated genome completeness by CheckM based on the presence of essential single copy genes (%). **Contamination** is the estimated genome contamination by CheckM based on the presence of duplicated single copy genes (%).The sequences can be found at NCBI GenBank.

Table S3. All regulated proteins (Mass Spectrometry).

UniProt ID	Gene	Lb21/Lb23 ratio	log2 (ratio)	Qvalue	Protein description
A0A131MBB2;Q18916	acs-22	4,68	2,23	4,26E-02	Fatty Acid CoA Synthetase family
Q9XWZ2;Q9XWZ2-2	acdh-11	2,14	1,09	2,17E-02	Acyl-CoA dehydrogenase family member 11;Isoform b of Acyl-CoA dehydrogenase family member 11
H2KY95;Q9TZ51	far-7	2,05	1,04	2,10E-02	Fatty Acid/Retinol binding protein
A9Z1L1;G5EBT3	srp-7	1,99	0,99	5,18E-06	SeRPin
Q8IG67	let-611	1,97	0,98	3,94E-02	Uncharacterized protein
O44501	fnta-1	1,89	0,91	4,77E-02	FarNesylTransferase, Alpha subunit
Q17761	T25B9.9	1,88	0,91	8,30E-04	6-phosphogluconate dehydrogenase, decarboxylating
P05690	vit-2	1,88	0,91	4,80E-256	Vitellogenin-2
Q9N4J2	vit-3	1,83	0,87	1,84E-04	Vitellogenin-3
O02108;Q336L8	cdc-37	1,77	0,82	3,13E-02	Probable Hsp90 co-chaperone cdc37;Cell Division Cycle related
P18948;P18948-2	vit-6	1,77	0,82	1,17E-231	Vitellogenin-6;Isoform a of Vitellogenin-6
O17365	srp-2	1,73	0,79	7,78E-03	SeRPin
Q17334	sodh-1	1,69	0,76	3,25E-08	Alcohol dehydrogenase 1
G5ECL3	prp-21	1,67	0,74	2,43E-02	Pre-RNA processing 21
O18693	acs-2	1,65	0,72	1,49E-03	Fatty Acid CoA Synthetase family
Q22492	hex-1	1,63	0,71	4,42E-06	Beta-hexosaminidase A
O17795;Q17703	stdh-2;stdh-1	1,61	0,69	1,14E-02	Putative steroid dehydrogenase 2;Putative steroid dehydrogenase 1
Q03577	dars-1	1,61	0,68	2,01E-02	Aspartate--tRNA ligase, cytoplasmic
A0A0K3AVS5	CELE_M02H5.8	1,59	0,67	4,66E-02	Uncharacterized protein
Q9GZE9	ldp-1	1,53	0,62	1,96E-06	Lipid droplet localized protein
G5EBF3	pud-2.1	1,50	0,58	1,75E-05	Protein Up-regulated in Daf-2(Gf)
Q22129	CELE_T03G6.3	0,65	-0,62	4,43E-02	Uncharacterized protein
G8JY94;Q21067;Q21067-2	ifc-2	0,64	-0,65	3,86E-03	Intermediate Filament, C;Intermediate filament protein ifc-2;Isoform b of Intermediate filament protein ifc-2
Q22169	trap-2	0,63	-0,66	3,78E-02	Translocon-associated protein subunit beta
Q9XWL1	ttr-17	0,63	-0,67	1,87E-03	TransThyretin-Related family domain
G1K0V9	iff-1	0,62	-0,68	3,23E-02	Initiation Factor Five (eIF-5A) homolog
Q22763	nep-22	0,61	-0,70	7,02E-05	NEPrilysin metallopeptidase family
Q19782	ifd-2	0,61	-0,72	1,29E-06	Intermediate filament protein ifd-2
O44985	teg-4	0,59	-0,77	3,50E-02	Tumorous Enhancer of Glp-1(Gf)
Q9TXK1	CELE_F54E2.1	0,58	-0,78	1,43E-02	Uncharacterized protein
M1Z854;Q19286	ifb-2	0,57	-0,80	1,38E-17	Intermediate Filament, B;Intermediate filament protein ifb-2
A0A1T5HUZ1;G5EEI5;H9G348;H9G349;H9G350;H9G351;H9G352;H9G353;H9G354;Q06561;Q06561-2;Q06561-4;Q06561-5	unc-52	0,57	-0,81	2,38E-02	Uncharacterized protein;Basement membrane proteoglycan;Isoform a, c, f of Basement membrane proteoglycan
O02252	pcp-3	0,55	-0,87	1,30E-02	Prolyl Carboxy Peptidase like
Q20660;Q7JP52	hsp-17	0,54	-0,90	3,96E-05	Heat Shock Protein
Q9XWU2	sup-1	0,52	-0,93	1,30E-02	Protein SUP-1
Q564Q1;Q564Q1-2	gale-1	0,52	-0,93	7,58E-03	UDP-glucose 4-epimerase;Isoform a of UDP-glucose 4-epimerase
P34528	K12H4.7	0,49	-1,03	3,78E-02	Putative serine protease K12H4.7
Q21750	aagr-2	0,48	-1,06	3,36E-02	Acid Alpha Glucosidase Relate
F5GUD3	tre-3	0,48	-1,07	4,06E-03	Trehalase
Q86NE0	asp-2	0,46	-1,12	5,44E-04	ASpartyl Protease
Q19876	thn-2	0,44	-1,17	6,39E-04	THaumatiN family
Q23683	CELE_ZK970.7	0,44	-1,17	2,96E-06	Uncharacterized protein
O45444	clec-63	0,44	-1,17	2,55E-09	C-type LECtin
Q18577	C42D4.1	0,43	-1,22	3,42E-16	Uncharacterized protein
O76836;Q2V4T0	CELE_T19D12.4	0,42	-1,24	5,41E-10	Uncharacterized protein
P43508	cpr-4	0,42	-1,24	3,13E-03	Cathepsin B-like cysteine proteinase 4
Q9BKP8	C17F4.7	0,41	-1,28	1,11E-02	Uncharacterized protein
A0A131MBC3;A0A131MCY4;G5EER7;G5EG67	tag-10	0,41	-1,28	5,38E-06	Uncharacterized protein
Q09365	ZK1320.3	0,41	-1,29	4,74E-03	Uncharacterized protein ZK1320.3
P55956	asp-3	0,39	-1,36	6,88E-03	Aspartic protease 3
Q86FL8	spp-5	0,38	-1,38	1,08E-04	SaPosin-like Protein family
O76672	CELE_H34I24.2	0,38	-1,41	1,93E-02	Uncharacterized protein
Q18020	hrg-7	0,36	-1,46	9,48E-03	Heme Responsive protein
Q7YTR9	C27B7.9	0,35	-1,50	1,07E-03	Uncharacterized protein
O01532	asp-5	0,34	-1,55	2,38E-03	ASpartyl Protease
G5EEI4	asp-1	0,34	-1,56	3,51E-09	ASpartyl Protease
Q2XN18	spp-14	0,33	-1,61	5,10E-05	SaPosin-like Protein family
A0A0K3ARI2;Q9XUF9;U4PBX8	C49C3.4	0,32	-1,63	1,94E-03	Uncharacterized protein
A0A0K3AVF2;O61874	dod-19	0,32	-1,64	1,02E-06	Downstream Of DAF-16 (Regulated by DAF-16)
O17038	col-143	0,29	-1,78	1,43E-02	COLlagen
Q5WRM0;Q9XUT9	CELE_K06G5.1	0,27	-1,86	1,83E-05	Uncharacterized protein
O45443	clec-65	0,18	-2,46	3,64E-02	C-type LECtin
O01530	asp-6	0,14	-2,88	5,05E-17	Aspartic protease 6

UniProt ID	Gene	Lb21/OP 50 ratio	log2 (ratio)	Qvalue	Protein description
H2KZ06;H2KZ07	clec-266	24,78	4,63	2,19E-10	C-type LECtin
Q11174	cht-1	13,52	3,76	1,83E-05	Probable endochitinase
Q22719;Q7JLZ9	CELE_T24B8.3	9,29	3,22	1,71E-04	Uncharacterized protein
G5EE48	ttr-50	8,93	3,16	1,25E-04	TransThyretin-Related family domain
Q17572	C01G6.3	8,47	3,08	1,30E-03	Uncharacterized protein
Q17334	sodh-1	7,50	2,91	2,10E-30	Alcohol dehydrogenase 1

UniProt ID	Gene	Lb21/OP 50 ratio	log2 (ratio)	Qvalue	Protein description
J7S164;J7SA65;P09588;Q27485	his-57;his-57; his-3;his-35	6,12	2,61	1,83E-18	Histone H2A
P04255;Q27484;Q27876;Q27894	his-11;his-41; his-48;his-4	6,02	2,59	1,37E-20	Histone H2B 1;Probable histone H2B 3;Probable histone H2B 4;Histone H2B 2
G5EFP4	sym-1	5,82	2,54	8,73E-04	SYM-1 protein
O18693	acs-2	5,58	2,48	3,94E-05	Fatty Acid CoA Synthetase family
K7ZUH9;P08898;Q10453;Q9U281	his-40;his-2; his-71;his-72	5,51	2,46	6,46E-07	Histone H3;Histone H3;Histone H3.3 type 1; Histone H3.3 type 2
Q9BL39	swsn-3	5,37	2,43	1,50E-02	SWI/SNF nucleosome remodeling complex component
P10771	his-24	5,37	2,42	4,00E-04	Histone H1.1
D5MCQ1;D5MCQ2;D5MCQ3;D5MCQ5;D5MCQ6;D5MCQ7;D5MCQ9;D5MCR3;D5MCR4;Q21900;Q7JMB8	sup-26	5,35	2,42	1,20E-03	SUPpressor
O45783	CELE_T12D8.5	5,29	2,40	1,79E-05	Uncharacterized protein
A7DTF0;Q9XWW2	mrg-1	4,97	2,31	2,69E-02	MRG (Mortality factor-Related Gene) related
Q94051	cav-1	4,76	2,25	3,36E-03	Caveolin-1
P62784	his-1	4,62	2,21	4,11E-16	Histone H4
P42170	rnr-2	4,53	2,18	2,20E-04	Ribonucleoside-diphosphate reductase small chain
Q19200	sto-1	4,37	2,13	5,21E-03	Stomatin-1
Q20684	lec-2	4,31	2,11	2,78E-17	GaLECtin
Q9N5F6	CELE_R193.2	4,25	2,09	3,84E-06	Uncharacterized protein
Q27511	htz-1	4,09	2,03	6,32E-05	Histone H2A.V
G5EEL9	hmg-11	4,05	2,02	8,10E-03	HMG
Q9XUR6	CELE_T05E12.3	3,95	1,98	4,58E-05	Uncharacterized protein
Q967F4;Q967F4-2;Q967F4-3; Q967F4-4	hmr-1	3,66	1,87	3,61E-03	Cadherin-related hmr-1;Isoform a, c, d of Cadherin-related hmr-1
P34345	gsto-1	3,61	1,85	1,56E-03	Glutathione transferase omega-1
G5EFV4	hmg-1.1	3,56	1,83	6,47E-04	HMG
Q9XVS3	clec-87	3,36	1,75	2,54E-03	C-type lectin domain-containing protein 87
O45577	cpf-2	3,13	1,64	8,39E-03	Cleavage and Polyadenylation Factor
G5EET2	dut-1	3,11	1,64	7,04E-03	DeoxyUTPase
Q966L5	E01A2.2	3,10	1,63	7,35E-03	Serrate RNA effector molecule homolog
Q22492	hex-1	2,98	1,58	7,24E-18	Beta-hexosaminidase A
A0A168H9W7;O61907;Q76NP4; Q86S28	lin-40	2,98	1,58	3,38E-02	Uncharacterized protein
Q19537	rpa-1	2,88	1,53	4,58E-05	Probable replication factor A 73 kDa subunit
Q9N363	dcap-1	2,85	1,51	4,63E-02	mRNA DeCAPping enzyme
O17406	attf-2	2,81	1,49	8,73E-09	AT hook Transcription Factor family
G5EC23	hcf-1	2,81	1,49	1,47E-02	HCF1 related
O01615	T19H12.2	2,76	1,46	3,83E-05	Acidic leucine-rich nuclear phosphoprotein 32-related protein 2
P90916	lin-53	2,75	1,46	1,20E-04	Probable histone-binding protein lin-53
Q4W4Y5;Q8MXD9	CELE_E02D9.1	2,75	1,46	2,74E-05	Uncharacterized protein
P91491	CELE_T23B3.2	2,74	1,45	7,09E-03	Uncharacterized protein
O76725	ctsa-2	2,74	1,45	4,60E-02	Carboxypeptidase
Q9N3F7	CELE_ Y53G8AR.9	2,69	1,43	9,05E-03	Uncharacterized protein
O17695	hda-1	2,69	1,43	4,21E-02	Histone deacetylase 1
O17687	nasp-2	2,65	1,41	1,73E-22	NASP (Human Nuclear Autoantigenic Sperm Protein) homolog
O02482	unc-37	2,65	1,41	4,30E-02	Transcription factor unc-37
G5EGA6	dhrs-4	2,61	1,38	1,14E-03	Dehydrogenase/reductase SDR family member 4
Q19326	unc-85	2,56	1,35	9,15E-04	Probable histone chaperone asf-1
H2KYN6;H2KYN7	smk-1	2,53	1,34	2,80E-02	SMEK (Dictyostelium Suppressor of MEK null) homolog
G1K0X0;Q1ZXU6; Q22655;Q22656	pgp-6;pgp-6; pgp-7;pgp-6	2,50	1,32	2,58E-02	p-GlycoProtein related
P91027	chdp-1	2,48	1,31	1,08E-11	Calponin Homology Domain containing Protein
O01971	emr-1	2,41	1,27	1,30E-03	Emerin homolog 1
Q94269	CELE_K10C2.1	2,41	1,27	2,05E-10	Carboxypeptidase
G5EE72	mrp-5	2,39	1,26	2,50E-03	Uncharacterized protein
Q18212	hel-1	2,36	1,24	2,33E-21	Spliceosome RNA helicase DDX39B homolog
O02115	pcn-1	2,29	1,19	6,58E-04	Proliferating cell nuclear antigen
Q9XVS2	sup-46	2,26	1,18	1,94E-03	SUPpressor
P55853	smo-1	2,25	1,17	1,29E-07	Small ubiquitin-related modifier
O17626	C31C9.2	2,24	1,16	1,29E-13	Uncharacterized protein
M1Z854;Q19286	ifb-2	2,22	1,15	8,79E-14	Intermediate Filament, B; Intermediate filament protein ifb-2
Q21702	CELE_R04B5.5	2,21	1,15	1,02E-02	Uncharacterized protein
G5EF87;H8ESF3	swsn-1	2,21	1,14	1,72E-04	SWI/SNF nucleosome remodeling complex component
Q20790	CELE_F54F7.3	2,18	1,12	1,30E-02	Uncharacterized protein
Q18599	acer-1	2,18	1,12	2,99E-24	ACEtyl-CoA Regulator
Q9XXI9	mcm-2	2,17	1,11	1,44E-02	DNA helicase
Q10661	dpy-30	2,14	1,09	1,51E-02	Dosage compensation protein dpy-30
Q21443	lmn-1	2,13	1,09	5,02E-19	Lamin-1
Q9NA74	CELE_Y57A10A.26	2,11	1,08	1,75E-05	Uncharacterized protein
Q9XVR7	mcm-3	2,10	1,07	6,48E-05	DNA helicase
Q965V4	xpo-2	2,10	1,07	8,01E-04	EXPOrtin (Nuclear export receptor)
P34328	hsp-12.2	2,08	1,06	9,79E-05	Heat shock protein Hsp-12.2
G5EDQ4;G5EDV5; G5EF89;G5EGA4	cle-1	2,07	1,05	6,06E-03	CLE-1C protein;CLE-1B protein; CoLlagens with Endostatin domain
O45599	cbd-1	2,07	1,05	7,11E-06	Chitin-binding domain protein cbd-1
G5ECY0	dlg-1	2,06	1,04	9,05E-03	Disks large homolog 1
Q22763	nep-22	2,04	1,03	1,71E-05	NEPrilysin metallopeptidase family

UniProt ID	Gene	Lb21/OP 50 ratio	log2 (ratio)	Qvalue	Protein description
Q21832	rnp-4	2,03	1,02	3,90E-05	RNA-binding protein 8A
D7SFI3	C02D5.4	2,02	1,01	5,43E-07	Uncharacterized protein
Q9N4L9	CELE_ZK973.9	2,01	1,01	2,30E-02	Uncharacterized protein
H2KYV3;Q18076	C18B2.5	2,01	1,01	6,14E-04	Uncharacterized protein
Q95ZI6	ZK353.9	2,01	1,01	4,96E-02	PITH domain-containing protein ZK353.9
Q23463	eftu-2	2,01	1,00	2,71E-03	Elongation Factor TU family
O44565	lam-1	1,98	0,99	1,24E-03	LAMinin related. See also lmb
O76565	F13C5.5	1,98	0,99	1,84E-02	Uncharacterized protein F13C5.5
Q18529	C39D10.7	1,98	0,98	1,36E-12	Uncharacterized protein
G5EBP5	CELE_ZC247.1	1,97	0,98	1,67E-04	Uncharacterized protein
Q20264	acs-11	1,97	0,98	9,05E-05	Fatty Acid CoA Synthetase family
O16214	CELE_F17A9.4	1,96	0,97	5,08E-04	Uncharacterized protein
P49029	mag-1	1,96	0,97	4,55E-05	Protein mago nashi homolog
Q18211	ran-3	1,95	0,97	2,13E-04	Regulator of chromosome condensation
Q21929	dhs-21	1,95	0,96	2,32E-07	L-xylulose reductase
Q9BKP3;Q9UB28;Q9UB29	let-805	1,94	0,96	3,86E-02	Uncharacterized protein;Myotactin form B;Myotactin form A
O44444	C02B10.4	1,94	0,95	6,38E-03	Uncharacterized protein
O45405	exl-1	1,93	0,95	1,43E-02	Chloride intracellular channel exl-1
Q4R127	CELE_K11D12.13	1,93	0,95	4,89E-02	Uncharacterized protein
O17389	tth-1	1,92	0,94	5,11E-06	Thymosin beta
P34685	cap-1	1,91	0,94	7,99E-03	F-actin-capping protein subunit alpha
A0A0K3AXF6;Q9U2U0	uaf-2	1,91	0,94	4,58E-03	U2AF splicing factor
A0A131MBC3;A0A131MCY4; G5EER7;G5EG67	tag-10	1,91	0,93	1,76E-03	Uncharacterized protein
O17915	ran-1	1,91	0,93	1,12E-05	GTP-binding nuclear protein ran-1
Q10663	icl-1	1,91	0,93	2,65E-47	Bifunctional glyoxylate cycle protein
Q9NEW6	rsp-3	1,90	0,93	6,65E-03	Probable splicing factor, arginine/serine-rich 3
G5EBJ8;P05634;P53017;P53018; P53019;Q17856;Q18461; Q21244;Q23519	CELE_Y59E9AR.1; msp-10;msp-19; msp-32;msp-33; msp-55;msp-49; msp-63;msp-152	1,89	0,92	1,17E-05	Major sperm protein;Major sperm protein 10/36/56/76/19/31/40/ 45/50/51/53/59/61/65/81/ 113/14/32/33/55/57/49/63/152
Q09665	tnc-2	1,88	0,91	2,23E-03	Troponin C, isoform 2
Q9NLD1	hrp-2	1,87	0,91	5,55E-12	HnRNP A1 homolog
Q23614	CELE_ZK822.2	1,87	0,91	2,72E-02	Uncharacterized protein
O44985	teg-4	1,86	0,90	2,49E-02	Tumorous Enhancer of Glp-1(Gf)
A9Z1L1;G5EBT3	srp-7	1,85	0,89	1,60E-03	SeRPin
P91423	CELE_T03F1.11	1,85	0,89	1,79E-03	Uncharacterized protein
Q10051	prp-19	1,84	0,88	2,36E-05	Pre-mRNA-processing factor 19
Q20616	skpo-1	1,84	0,88	4,11E-04	Peroxidase skpo-1
Q18817	ben-1	1,84	0,88	9,79E-05	Tubulin beta chain
Q9XUW5	ddx-17	1,83	0,87	2,24E-02	DEAD boX helicase homolog
G5EET8	pud-1.2	1,83	0,87	1,14E-06	Protein Up-regulated in Daf-2(Gf)
G5ECR7	elb-1	1,82	0,87	4,56E-02	ELongin B
G5EE12	cash-1	1,81	0,86	3,81E-02	CKA And Striatin Homolog
Q17886	nasp-1	1,80	0,85	2,65E-03	NASP (Human Nuclear Autoantigenic Sperm Protein) homolog
P34556	cdk-1	1,80	0,85	6,83E-03	Cyclin-dependent kinase 1
Q05062	cdc-42	1,79	0,84	1,03E-02	Cell division control protein 42 homolog
O61787	inx-16	1,78	0,83	2,19E-02	Innexin-16
P17343	gpb-1	1,77	0,82	1,59E-04	Guanine nucleotide-binding protein subunit beta-1
Q09967	egg-1	1,76	0,82	4,25E-02	LDL receptor repeat-containing protein egg-1
Q23089	xpo-1	1,75	0,81	4,95E-02	EXPOrtin (Nuclear export receptor)
O76836;Q2V4T0	CELE_T19D12.4	1,75	0,81	1,08E-03	Uncharacterized protein
Q27249	lev-11	1,75	0,80	1,58E-07	Tropomyosin isoforms c/e
G5EBK3	erm-1	1,74	0,80	4,37E-08	ERM-1B
Q20173	twf-2	1,74	0,80	7,68E-03	TWinFilin actin binding protein homolog
H9G2S0;Q19191	CELE_F08B12.4; F08B12.4	1,74	0,80	1,20E-07	Uncharacterized protein; Uncharacterized protein F08B12.4
Q69ZI6	CELE_T12D8.10	1,72	0,79	1,88E-02	Uncharacterized protein
Q9U2F2	cpt-1	1,72	0,78	1,37E-03	Carnitine Palmitoyl Transferase
O45812	CELE_T23G11.7	1,72	0,78	4,99E-02	Uncharacterized protein
G5EFE3	CELE_H03A11.2	1,71	0,77	5,73E-09	Uncharacterized protein
Q21763	CELE_R05H5.3	1,71	0,77	2,69E-06	Uncharacterized protein
Q9GZH2	ruvb-2	1,70	0,76	9,88E-03	RuvB-like 2
Q21295;Q9U3B9;Q9U3C1	npp-1	1,69	0,76	5,00E-02	Nuclear Pore complex Protein
G5ECV9	alh-3	1,69	0,76	1,99E-06	10-formyltetrahydrofolate dehydrogenase
P34650	ZK632.4	1,69	0,76	5,92E-03	Probable mannose-6-phosphate isomerase
Q10020	T28D9.1	1,69	0,75	2,83E-02	Uncharacterized protein T28D9.1
N1NTN5;Q17971	C14C10.5	1,67	0,74	3,41E-02	Uncharacterized protein
Q9XWP0	mrps-28	1,67	0,74	9,36E-03	Mitochondrial Ribosomal Protein, Small
K8ESM2	CELE_Y16B4A.2	1,67	0,74	6,34E-03	Carboxypeptidase
P49595;Q8MNS3	F42G9.1; CELE_F42G9.1	1,67	0,74	3,57E-02	Probable protein phosphatase 2C F42G9.1; Uncharacterized protein
P12845	myo-2	1,67	0,74	1,75E-14	Myosin-2
Q20507	acbp-3	1,65	0,72	1,93E-03	Acyl-CoA-binding protein homolog 3
O17607	ruvb-1	1,65	0,72	3,23E-02	RuvB-like 1
Q22799	dlc-1	1,64	0,71	9,50E-06	Dynein light chain 1, cytoplasmic
Q19459;Q19459-2	F14E5.2	1,63	0,70	2,76E-02	Golgi apparatus protein 1 homolog; Isoform b of Golgi apparatus protein 1 homolog
Q20222;Q27GU2	lbp-3	1,63	0,70	1,07E-03	Fatty acid-binding protein homolog 3;Lipid Binding Protein
G4SL51	pqn-52	1,63	0,70	1,43E-02	Prion-like-(Q/N-rich)-domain-bearing protein
Q9GPA1	CELE_F48E3.4	1,62	0,70	1,44E-06	Uncharacterized protein
Q5WRT8;Q5WRT9	npp-21	1,62	0,70	2,88E-02	Nuclear Pore complex Protein
Q8MXS9;Q9N3S3;Q9N3S4	rnp-6	1,62	0,70	4,05E-02	RNP (RRM RNA binding domain) containing

UniProt ID	Gene	Lb21/OP 50 ratio	log2 (ratio)	Qvalue	Protein description
Q9TXH9	ubxn-1	1,62	0,70	3,03E-02	UBX domain-containing protein 1
O16297	mcm-7	1,62	0,69	1,36E-03	DNA replication licensing factor MCM7
G8XYY6;Q23232	wdr-4	1,61	0,69	1,80E-05	tRNA (guanine-N(7)-)-methyltransferase non-catalytic subunit
P90900	ifa-4	1,61	0,69	1,58E-04	Intermediate filament protein ifa-4
A0A061AD21;A0A061AJI9;C1P640; C1P641;G5EEV6;Q21313	epi-1	1,61	0,68	4,47E-06	Uncharacterized protein;Uncharacterized protein; Uncharacterized protein;Uncharacterized protein; Laminin alpha;Laminin-like protein epi-1
Q20655	ftt-2	1,61	0,68	1,07E-10	14-3-3-like protein 2
O01806	ssb-1	1,60	0,68	1,35E-02	Sjogren Syndrome antigen B homolog
Q9XTT9	rpt-6	1,60	0,68	7,95E-03	Proteasome Regulatory Particle, ATPase-like
Q18100;Q4PIU9; Q53U86;Q53U87	sax-7	1,59	0,67	3,13E-02	Sensory AXon guidance;Sensory AXon guidance;SAX-7 SHORTFORM;SAX-7 LONGFORM
Q9BI71;Q9BI72	epn-1	1,59	0,67	4,41E-02	EPsiN (Endocytic protein) homolog
Q95PZ7	CELE_Y66D12A.9	1,59	0,67	1,79E-02	Uncharacterized protein
Q22752	dyrb-1	1,58	0,66	1,74E-03	Dynein light chain roadblock
H2KZK7;Q965K3	dpf-3	1,57	0,66	3,42E-02	Dipeptidyl Peptidase Four (IV) family
Q03565	baf-1	1,57	0,65	3,17E-03	Barrier-to-autointegration factor 1
I2HAG1;Q09581;Q95ZI4	lec-3	1,57	0,65	4,93E-04	Galectin;32 kDa beta-galactoside-binding lectin lec-3;Galectin
Q9U1W8	lsm-3	1,57	0,65	2,88E-02	LSM Sm-like protein
O44887	inx-13	1,55	0,63	1,35E-02	Innexin
P19826;Q8MPS2	deb-1	1,54	0,63	7,11E-06	Vinculin;DEnse Body
Q19775;Q19775-4; Q19775-5	ppm-1	1,54	0,63	1,38E-04	Protein phosphatase ppm-1;Isoform b of Protein phosphatase ppm-1;Isoform c of Protein phosphatase ppm-1
Q9N3G0	CELE_Y53G8AR.6	1,54	0,63	2,13E-02	Uncharacterized protein
P90992	misc-1	1,54	0,63	9,91E-03	Mitochondrial 2-oxoglutarate/malate carrier protein
Q9TYW1	vha-19	1,54	0,62	3,64E-03	Vacuolar H ATPase
G5ECG8;G5EEC4; G5EFK7;G5EFW2	vab-10	1,54	0,62	1,22E-07	Uncharacterized protein;VAB-10A protein;Uncharacterized protein;Uncharacterized protein
O44781;Q9BL61	CELE_F33D11.10; CELE_Y65B4A.6	1,54	0,62	7,86E-05	Uncharacterized protein
G5EBF3	pud-2.1	1,54	0,62	7,56E-05	Protein Up-regulated in Daf-2(Gf)
G5EC91	dpy-11	1,53	0,61	4,32E-03	DumPY: shorter than wild-type
P34369	prp-8	1,52	0,61	5,23E-04	Pre-mRNA-splicing factor 8 homolog
P48727	gsp-2	1,52	0,61	1,19E-03	Serine/threonine-protein phosphatase PP1-beta
Q09422	pgam-5	1,52	0,61	4,04E-02	Serine/threonine-protein phosphatase Pgam5, mitochondrial
Q22037;Q22037-4	hrp-1	1,51	0,60	3,83E-05	Heterogeneous nuclear ribonucleoprotein A1;Isoform d of Heterogeneous nuclear ribonucleoprotein A1
G5EEL1;G5EFG1;G5EFL5	alp-1	1,50	0,58	1,50E-03	ALP/Enigma encoding
Q18823	lam-2	1,50	0,58	2,61E-05	Laminin-like protein lam-2
Q11176	unc-78	1,50	0,58	6,36E-04	Actin-interacting protein 1
Q21284	CELE_K07E3.4	1,50	0,58	5,76E-09	Uncharacterized protein
O01868	rpl-24.1	0,67	-0,58	5,78E-03	60S ribosomal protein L24
B7WNA0	pyk-1	0,67	-0,58	8,06E-11	Pyruvate kinase
O61790	R12E2.11	0,67	-0,59	1,63E-02	Orotate phosphoribosyltransferase
Q21770	wago-1	0,66	-0,59	3,23E-04	Argonaute protein wago-1
H2L0M0;Q9GZI3	pod-2	0,66	-0,60	2,27E-02	Uncharacterized protein
O44512	isp-1	0,66	-0,60	2,92E-03	Cytochrome b-c1 complex subunit Rieske, mitochondrial
Q7Z1Q3	alh-12	0,66	-0,60	7,37E-08	ALdehyde deHydrogenase
O02089	msra-1	0,65	-0,62	1,21E-04	Methionine Sulfoxide Reductase A
G5EDZ7	cdr-4	0,65	-0,63	6,21E-03	CaDmium Responsive
O61235	ctl-1	0,64	-0,64	4,36E-09	Catalase-2
O17622	C29F7.3	0,64	-0,64	2,15E-02	UMP-CMP kinase 1
Q10121	C23G10.2	0,64	-0,65	2,55E-02	RutC family protein C23G10.2
O02108;Q336L8	cdc-37	0,63	-0,66	4,13E-02	Probable Hsp90 co-chaperone cdc37;Cell Division Cycle related
G5ED31;G5EDD1	ucr-2.1	0,63	-0,67	4,58E-05	Ubiquinol-Cytochrome c oxidoReductase complex
Q8MXI1	ucr-11	0,62	-0,68	2,20E-03	Ubiquinol-Cytochrome c oxidoReductase complex
Q9XWU9	CELE_Y37D8A.19	0,62	-0,69	9,43E-04	Uncharacterized protein
Q9XVF7	rpl-2	0,62	-0,69	6,88E-06	60S ribosomal protein L8
Q9XW17	car-1	0,62	-0,69	4,11E-11	Cytokinesis, Apoptosis, RNA-associated
Q9N5E4	CELE_T02H6.11	0,62	-0,70	5,33E-04	Uncharacterized protein
Q09975	lys-8	0,62	-0,70	3,42E-02	LYSozyme
P53585	acly-1	0,62	-0,70	1,01E-03	Probable ATP-citrate synthase
Q9XXR4	ttr-24	0,62	-0,70	2,25E-04	TransThyretin-Related family domain
C1P622;Q09517;S6FCZ1	let-767	0,61	-0,72	1,47E-04	Uncharacterized protein;Very-long-chain 3-oxooacyl-coA reductase let-767;Uncharacterized protein
G5EET3	cdr-6	0,61	-0,72	1,20E-02	CaDmium Responsive
Q9N4D8	nlp-40	0,61	-0,72	3,67E-02	Neuropeptide-like peptides nlp-40
O62146	F09B12.3	0,61	-0,72	9,41E-03	Putative phospholipase B-like 2
C6KRL2	CELE_F23D12.11	0,60	-0,73	3,00E-02	Uncharacterized protein
Q23068	CELE_W01A11.1	0,60	-0,73	4,07E-05	Epoxide hydrolase
A5JYX5-2	dhs-3	0,60	-0,74	5,33E-07	Isoform a of Protein dhs-3
H2KZG6	acdh-1	0,59	-0,75	2,99E-02	Acyl CoA DeHydrogenase
O44400	F37C4.5	0,59	-0,75	6,89E-31	Protein F37C4.5
G5EEH6	ivd-1	0,59	-0,76	5,61E-06	Isovaleryl-CoA dehydrogenase
Q18853	cyc-1	0,59	-0,77	3,91E-04	CYtochrome C
Q9GUF2	acp-6	0,59	-0,77	2,56E-09	ACid Phosphatase family
P34559	ech-6	0,59	-0,77	1,13E-13	Probable enoyl-CoA hydratase, mitochondrial
G5ECT2	CELE_F15E6.3	0,58	-0,79	2,80E-02	RNA binding protein
O18000	pes-9	0,58	-0,79	2,74E-07	Patterned Expression Site
O17271	heh-1	0,57	-0,80	1,07E-03	Putative protein heh-1
Q22968	gcst-1	0,57	-0,80	2,83E-06	Aminomethyltransferase

UniProt ID	Gene	Lb21/OP 50 ratio	log2 (ratio)	Qvalue	Protein description
O17680	sams-1	0,57	-0,80	3,98E-19	Probable S-adenosylmethionine synthase 1
Q9Y041	hgo-1	0,57	-0,81	9,86E-04	Homogentisate 1,2-dioxygenase
P54688	bcat-1	0,57	-0,81	3,12E-02	Branched-chain-amino-acid aminotransferase, cytosolic
Q21962;Q86LS6	gldc-1	0,57	-0,81	1,33E-06	Glycine cleavage system P protein
Q22993	pmt-2	0,57	-0,81	1,84E-11	Phosphoethanolamine MethylTransferase
Q20829	aman-1	0,57	-0,82	1,22E-02	Alpha-mannosidase
P34466	clu-1	0,57	-0,82	1,07E-03	Clustered mitochondria protein homolog
Q17339	gld-1	0,56	-0,83	4,31E-02	Female germline-specific tumor suppressor gld-1
P52713	alh-8	0,56	-0,83	1,26E-16	Probable methylmalonate-semialdehyde dehydrogenase [acylating], mitochondrial
Q18683	C47E12.2	0,56	-0,84	4,98E-02	Uncharacterized protein
Q9U2M4	hphd-1	0,56	-0,84	2,27E-11	Hydroxyacid-oxoacid transhydrogenase, mitochondrial
A0A0K3ASN4;A0A0K3AT80;A0A0K3AVI4;A0A0K3AW96;A0A0K3AYI1;D0VWL6;D0VWL7	fln-2	0,55	-0,86	2,46E-02	FiLamiN (Actin binding protein) homolog
Q19278;Q8MNT7	hach-1	0,55	-0,87	7,23E-12	3-hydroxyisobutyryl-CoA hydrolase, mitochondrial;3-hydroxyisobutyryl-CoA hydrolase, mitochondrial
G5EGP8	cpz-1	0,54	-0,89	4,46E-05	CathePsin Z
Q5WRM0;Q9XUT9	CELE_K06G5.1	0,54	-0,90	7,86E-03	Uncharacterized protein
Q8IG67	let-611	0,54	-0,90	4,08E-02	Uncharacterized protein
Q09652	gstk-1	0,53	-0,91	2,34E-02	Glutathione S-transferase kappa 1
Q9NAB4	gst-26	0,53	-0,91	4,32E-02	Glutathione S-Transferase
E9P851	mctp-1	0,53	-0,92	1,13E-02	Multiple C2 and Transmembrane region Protein family homolog
P34383	far-2	0,53	-0,92	1,06E-65	Fatty-acid and retinol-binding protein 2
Q22633	hpd-1	0,52	-0,93	5,02E-06	4-hydroxyphenylpyruvate dioxygenase
Q19246	dhs-25	0,52	-0,93	1,24E-04	DeHydrogenases, Short chain
P18948;P18948-2	vit-6	0,52	-0,94	0,00E+00	Vitellogenin-6;Isoform a of Vitellogenin-6
P24890	ctb-1	0,51	-0,97	4,54E-02	Cytochrome b
Q9N4X8	gst-10	0,51	-0,97	2,10E-04	Glutathione S-transferase P 10
P43508	cpr-4	0,51	-0,98	1,38E-03	Cathepsin B-like cysteine proteinase 4
G5EDV7	CELE_Y53H1B.2	0,51	-0,98	2,62E-03	Uncharacterized protein
P34689	glh-1	0,51	-0,98	5,39E-12	ATP-dependent RNA helicase glh-1
Q19876	thn-2	0,50	-1,00	9,27E-04	THaumatiN family
Q9NAB3	gst-27	0,50	-1,00	2,10E-02	Glutathione S-Transferase
O17345	ttr-6	0,50	-1,01	1,13E-04	TransThyretin-Related family domain
Q20898	ifet-1	0,49	-1,02	4,32E-02	Translational repressor ifet-1
A0A0K3AVF2;O61874	dod-19	0,49	-1,02	2,18E-03	Downstream Of DAF-16 (Regulated by DAF-16)
Q22768	CELE_T25B9.1	0,49	-1,04	2,22E-03	Uncharacterized protein
Q9N585	ppw-2	0,48	-1,05	8,93E-03	Piwi-like protein
Q22814	gst-13	0,48	-1,07	1,78E-02	Glutathione S-Transferase
Q95YF3	cgh-1	0,47	-1,08	6,78E-28	ATP-dependent RNA helicase cgh-1
I2HA98;P90925	pah-1	0,46	-1,11	5,25E-06	PhenylAlanine Hydroxylase;Probable phenylalanine-4-hydroxylase 1
Q18938	gst-42	0,46	-1,12	3,71E-02	Probable maleylacetoacetate isomerase
G5EC10	lec-9	0,45	-1,15	6,02E-09	Galectin
Q18040	oatr-1	0,45	-1,16	3,40E-16	Probable ornithine aminotransferase, mitochondrial
O44503;U4PFH6	aass-1	0,44	-1,17	1,05E-03	AminoAdipate-Semialdehyde Synthase homolog
G5ECL3	prp-21	0,44	-1,19	1,16E-02	Pre-RNA processing 21
P91398	cey-3	0,43	-1,21	4,84E-05	C. Elegans Y-box
Q23044	CELE_T22B7.7	0,43	-1,23	2,64E-03	Uncharacterized protein
Q93315	cytb-5.1	0,42	-1,26	8,51E-03	YTochrome B
Q20615	dod-23	0,40	-1,30	4,97E-02	Downstream Of DAF-16 (Regulated by DAF-16)
Q2XN18	spp-14	0,40	-1,34	8,49E-04	SaPosin-like Protein family
P19974	cyc-2.1	0,39	-1,34	1,46E-06	Cytochrome c 2.1
O45346	osm-11	0,39	-1,36	3,13E-06	Uncharacterized protein
Q19478	far-3	0,39	-1,37	3,25E-16	Fatty Acid/Retinol binding protein
P91306	cey-2	0,38	-1,41	1,99E-11	C. Elegans Y-box
Q9U3Q6	ugt-22	0,38	-1,41	1,29E-02	UDP-glucuronosyltransferase
Q19699	gfat-2	0,37	-1,43	4,43E-06	Glutamine-Fructose 6-phosphate AminoTransferase homolog
Q19130;Q95QM8	gfat-1	0,37	-1,44	1,62E-03	Glutamine-Fructose 6-phosphate AminoTransferase homolog
Q9N384	lec-6	0,37	-1,45	4,73E-16	Galectin
Q21750	aagr-2	0,36	-1,46	1,23E-02	Acid Alpha Glucosidase Relate
Q18026	flu-2	0,36	-1,49	5,72E-10	Kynureninase
P55956	asp-3	0,35	-1,50	5,77E-04	Aspartic protease 3
Q9BL43	CELE_Y71H2AM.13	0,35	-1,53	8,51E-05	Uncharacterized protein
P43510;Q8MQC6	cpr-6	0,34	-1,56	5,19E-13	Cathepsin B-like cysteine proteinase 6;Cysteine PRotease related
O17038	col-143	0,34	-1,57	3,73E-02	COLlagen
O02215	cbl-1	0,31	-1,68	3,14E-02	Cystathionine Beta Lyase
O01530	asp-6	0,28	-1,85	3,43E-13	Aspartic protease 6
Q09486	C30G12.2	0,27	-1,89	8,88E-03	Uncharacterized protein
Q09365	ZK1320.3	0,26	-1,93	1,70E-02	Uncharacterized protein ZK1320.3
H2KZV5	CELE_T21H3.1	0,26	-1,96	1,01E-04	Uncharacterized protein
P34528	K12H4.7	0,25	-1,99	1,38E-02	Putative serine protease K12H4.7
P90889	CELE_F55H12.4	0,24	-2,07	8,00E-08	Uncharacterized protein
O01532	asp-5	0,24	-2,08	4,24E-03	ASpartyl Protease
P55155	vit-1	0,24	-2,08	5,72E-11	Vitellogenin-1
P46562	alh-9	0,23	-2,09	1,63E-38	Putative aldehyde dehydrogenase family 7 member A1 homolog
Q7YTR9	C27B7.9	0,23	-2,11	8,60E-04	Uncharacterized protein

UniProt ID	Gene	Lb21/OP 50 ratio	log2 (ratio)	Qvalue	Protein description
G5EEI4	asp-1	0,22	-2,16	1,42E-08	ASpartyl Protease
Q86NE0	asp-2	0,20	-2,30	3,52E-07	ASpartyl Protease
Q20950	CELE_F57F5.1	0,20	-2,35	9,24E-03	Uncharacterized protein
P55216	cth-2	0,18	-2,43	2,99E-04	Putative cystathionine gamma-lyase 2
P06125	vit-5	0,13	-2,96	5,03E-61	Vitellogenin-5
Q86FL8	spp-5	0,13	-2,97	1,69E-06	SaPosin-like Protein family
P18947	vit-4	0,10	-3,29	8,72E-299	Vitellogenin-4
Q9N4J2	vit-3	0,10	-3,30	2,13E-04	Vitellogenin-3
O01467	C04E6.5	0,09	-3,51	2,66E-02	Uncharacterized protein
O17725	CELE_D1086.3	0,08	-3,58	1,07E-02	Uncharacterized protein

UniProt ID	Gene	Lb23/OP 50 ratio	log2 (ratio)	Qvalue	Protein description
H2KZ06;H2KZ07	clec-266	19,02	4,25	3,89E-11	C-type LECtin
Q11174	cht-1	16,46	4,04	1,16E-05	Probable endochitinase
G5EE48	ttr-50	8,54	3,09	1,08E-04	TransThyretin-Related family domain
Q22719;Q7JLZ9	CELE_T24B8.3	8,35	3,06	3,96E-04	Uncharacterized protein
Q17572	C01G6.3	7,48	2,90	1,37E-03	Uncharacterized protein
D5MCQ1;D5MCQ2;D5MCQ3; D5MCQ5;D5MCQ6;D5MCQ7; D5MCQ9;D5MCR3;D5MCR4;Q21900 ;Q7JMB8	sup-26	6,51	2,70	3,38E-04	SUPpressor
Q9N5F6	CELE_R193.2	6,14	2,62	1,93E-07	Uncharacterized protein
P04255;Q27484;Q27876;Q27894	his-11;his-41; his-48;his-4	5,95	2,57	1,01E-16	Histone H2B 1;Probable histone H2B 3;Probable histone H2B 4;Histone H2B 2
J7S164;J7SA65; P09588;Q27485	his-57;his-57; his-3;his-35	5,68	2,51	1,97E-15	Histone H2A
Q20684	lec-2	5,50	2,46	8,60E-22	GaLECtin
P10771	his-24	5,45	2,45	2,98E-04	Histone H1.1
Q967F4;Q967F4-2; Q967F4-3;Q967F4-4	hmr-1	5,19	2,38	1,88E-02	Cadherin-related hmr-1;Isoform a, c, d of Cadherin-related hmr-1
Q19200	sto-1	5,14	2,36	5,78E-03	Stomatin-1
Q94051	cav-1	5,00	2,32	4,71E-03	Caveolin-1
K7ZUH9;P08898; Q10453;Q9U281	his-40;his-2; his-71;his-72	4,97	2,31	2,68E-08	Histone H3;Histone H3;Histone H3.3 type 1;Histone H3.3 type 2
Q9BL39	swsn-3	4,82	2,27	3,11E-02	SWI/SNF nucleosome remodeling complex component
A0A131MBC3;A0A131MCY4; G5EER7;G5EG67	tag-10	4,64	2,21	7,09E-05	Uncharacterized protein
A7DTF0;Q9XWW2	mrg-1	4,57	2,19	1,94E-02	MRG (Mortality factor-Related Gene) related
Q17334	sodh-1	4,43	2,15	1,43E-22	Alcohol dehydrogenase 1
O76725	ctsa-2	4,35	2,12	2,20E-02	Carboxypeptidase
G5EC23	hcf-1	4,33	2,11	4,96E-02	HCF1 related
P42170	rnr-2	4,12	2,04	7,79E-04	Ribonucleoside-diphosphate reductase small chain
O76836;Q2V4T0	CELE_T19D12.4	4,12	2,04	1,10E-06	Uncharacterized protein
G5EFP4	sym-1	3,99	2,00	1,40E-03	SYM-1 protein
P62784	his-1	3,88	1,96	7,29E-12	Histone H4
M1Z854;Q19286	ifb-2	3,87	1,95	4,14E-21	Intermediate Filament, B;Intermediate filament protein ifb-2
Q27511	htz-1	3,73	1,90	1,75E-04	Histone H2A.V
Q966L5	E01A2.2	3,71	1,89	4,56E-03	Serrate RNA effector molecule homolog
Q9XUR6	CELE_T05E12.3	3,67	1,88	8,19E-05	Uncharacterized protein
P91491	CELE_T23B3.2	3,66	1,87	5,69E-03	Uncharacterized protein
O45783	CELE_T12D8.5	3,58	1,84	2,63E-06	Uncharacterized protein
Q9BIB7	hrpf-1	3,57	1,84	2,77E-02	HnRNP F homolog
G5EFV4	hmg-1.1	3,54	1,82	1,36E-03	HMG
Q95Y97	rpa-2	3,44	1,78	6,24E-03	Replication Protein A homolog
O18693	acs-2	3,38	1,76	2,64E-04	Fatty Acid CoA Synthetase family
O45444	clec-63	3,37	1,75	1,41E-16	C-type LECtin
G5EEL9	hmg-11	3,34	1,74	2,00E-02	HMG
Q22763	nep-22	3,33	1,73	4,16E-08	NEPrilysin metallopeptidase family
Q9XVS3	clec-87	3,31	1,73	2,00E-02	C-type lectin domain-containing protein 87
O17406	attf-2	3,31	1,73	9,52E-09	AT hook Transcription Factor family
Q9N3F7	CELE_Y53G8AR.9	3,19	1,67	9,06E-03	Uncharacterized protein
O44985	teg-4	3,17	1,66	8,26E-03	Tumorous Enhancer of Glp-1(Gf)
G5EET2	dut-1	3,10	1,63	4,04E-03	DeoxyUTPase
O76672	CELE_H34I24.2	3,03	1,60	4,89E-02	Uncharacterized protein
P91027	chdp-1	2,94	1,56	3,28E-13	Calponin Homology Domain containing Protein
Q09967	egg-1	2,87	1,52	3,55E-02	LDL receptor repeat-containing protein egg-1
O45405	exl-1	2,86	1,52	1,62E-02	Chloride intracellular channel exl-1
P90916	lin-53	2,85	1,51	2,41E-04	Probable histone-binding protein lin-53
O01615	T19H12.2	2,85	1,51	6,06E-05	Acidic leucine-rich nuclear phosphoprotein 32-related protein 2
Q94269	CELE_K10C2.1	2,83	1,50	2,34E-11	Carboxypeptidase
Q4W4Y5;Q8MXD9	CELE_E02D9.1	2,83	1,50	2,37E-06	Uncharacterized protein
A0A168H9W7;O61907;Q76NP4;Q86 S28	lin-40	2,80	1,49	4,21E-02	Uncharacterized protein
G5EGA6	dhrs-4	2,79	1,48	1,42E-04	Dehydrogenase/reductase SDR family member 4
Q19537	rpa-1	2,79	1,48	6,36E-06	Probable replication factor A 73 kDa subunit
Q9N363	dcap-1	2,74	1,45	8,44E-03	mRNA DeCAPping enzyme
P34345	gsto-1	2,69	1,43	1,18E-02	Glutathione transferase omega-1
Q9NA74	CELE_Y57A10A.26	2,66	1,41	2,80E-08	Uncharacterized protein
Q19326	unc-85	2,64	1,40	8,56E-03	Probable histone chaperone asf-1
P34328	hsp-12.2	2,60	1,38	6,90E-07	Heat shock protein Hsp-12.2
P91423	CELE_T03F1.11	2,60	1,38	4,53E-05	Uncharacterized protein
O17687	nasp-2	2,54	1,35	8,01E-21	NASP (Human Nuclear Autoantigenic Sperm Protein) homolog

UniProt ID	Gene	Lb23/OP 50 ratio	log2 (ratio)	Qvalue	Protein description
O02482	unc-37	2,54	1,34	7,26E-03	Transcription factor unc-37
Q9N4L9	CELE_ZK973.9	2,51	1,33	1,90E-02	Uncharacterized protein
G5EF87;H8ESF3	swsn-1	2,51	1,32	1,57E-04	SWI/SNF nucleosome remodeling complex component
G5EE72	mrp-5	2,50	1,32	3,75E-03	Uncharacterized protein
Q19555	scc-3	2,50	1,32	1,69E-02	Cohesin subunit scc-3
O17626	C31C9.2	2,48	1,31	5,08E-15	Uncharacterized protein
Q18212	hel-1	2,46	1,30	1,38E-17	Spliceosome RNA helicase DDX39B homolog
Q20790	CELE_F54F7.3	2,43	1,28	9,29E-03	Uncharacterized protein
Q27249	lev-11	2,41	1,27	3,63E-08	Tropomyosin isoforms c/e
Q09936	C53C9.2	2,37	1,24	3,53E-02	Uncharacterized protein C53C9.2
O61787	inx-16	2,37	1,24	3,64E-02	Innexin-16
Q19782	ifd-2	2,37	1,24	8,63E-05	Intermediate filament protein ifd-2
Q9BKP3;Q9UB28; Q9UB29	let-805	2,36	1,24	3,57E-02	Uncharacterized protein;Myotactin form B;Myotactin form A
Q9XVS2	sup-46	2,34	1,23	1,75E-04	SUPpressor
O02115	pcn-1	2,34	1,23	5,24E-04	Proliferating cell nuclear antigen
Q10661	dpy-30	2,34	1,23	3,13E-03	Dosage compensation protein dpy-30
Q7JKP2;Q9U362	pgrn-1	2,33	1,22	2,23E-02	ProGRaNulin homolog
Q21443	lmn-1	2,33	1,22	2,24E-31	Lamin-1
O01971	emr-1	2,32	1,22	2,66E-02	Emerin homolog 1
Q22799	dlc-1	2,30	1,20	8,53E-07	Dynein light chain 1, cytoplasmic
Q23463	eftu-2	2,30	1,20	1,96E-03	Elongation Factor TU family
Q09665	tnc-2	2,28	1,19	2,05E-03	Troponin C, isoform 2
Q8WQA4	exc-4	2,26	1,18	2,58E-02	Chloride intracellular channel exc-4
O17389	tth-1	2,25	1,17	1,35E-08	Thymosin beta
A0A1T5HUZ1;G5EEI5;H9G348;H9G349;H9G350;H9G351;H9G352;H9G353;H9G354;Q06561;Q06561-2;Q06561-4;Q06561-5	unc-52	2,21	1,14	1,91E-02	Uncharacterized proteins;Basement membrane proteoglycan;Isoform a, c, f of Basement membrane proteoglycan;
G5ECY0	dlg-1	2,20	1,14	4,85E-03	Disks large homolog 1
Q9XWL1	ttr-17	2,20	1,14	1,53E-03	TransThyretin-Related family domain
O45599	cbd-1	2,18	1,12	4,11E-07	Chitin-binding domain protein cbd-1
Q21832	rnp-4	2,18	1,12	1,50E-04	RNA-binding protein 8A
G4SL51	pqn-52	2,17	1,12	1,71E-03	Prion-like-(Q/N-rich)-domain-bearing protein
P90900	ifa-4	2,16	1,11	3,70E-09	Intermediate filament protein ifa-4
G5ECG8;G5EEC4; G5EFK7;G5EFW2	vab-10	2,15	1,11	3,93E-12	Uncharacterized protein;VAB-10A protein;Uncharacterized protein;Uncharacterized protein
P21137;P21137-10;P21137-11;P21137-12;P21137-13;P21137-2;P21137-3;P21137-4;P21137-5; P21137-6;P21137-7;P21137-8;P21137-9	kin-1	2,15	1,11	3,36E-02	cAMP-dependent protein kinase catalytic subunit;Isoform j, k, l, m, a, b, c, d, f, g, h, i of cAMP-dependent protein kinase catalytic subunit
G5EBP5	CELE_ZC247.1	2,15	1,11	1,02E-06	Uncharacterized protein
Q20660;Q7JP52	hsp-17	2,15	1,10	5,25E-07	Heat Shock Protein
G5EBJ8;P05634;P53017;P53018;P53019;Q17856;Q18461;Q21244;Q23519	CELE_Y59E9AR.1; msp-10;msp-19; msp-32;msp-33; msp-55;msp-49; msp-63; msp-152	2,14	1,10	1,96E-07	Major sperm protein;Major sperm protein 10/36/56/76;Major sperm protein19/31/40/45/50/51/53/59/61/65/81/113 /142/32/33/55/57/49/63/152
A0A061ACM2;A0A061ACP8; A0A061ADW6;G5ED33;G5EFE0;O18250;Q7YTG1	eps-8	2,14	1,10	3,94E-03	EPS (Human endocytosis) related
H2KYV3;Q18076	C18B2.5	2,13	1,09	3,31E-04	Uncharacterized protein
Q10051	prp-19	2,12	1,09	1,10E-05	Pre-mRNA-processing factor 19
O17921	tbb-1	2,11	1,08	1,82E-02	Tubulin beta chain
P55853	smo-1	2,10	1,07	6,65E-06	Small ubiquitin-related modifier
D7SFI3	C02D5.4	2,09	1,07	1,20E-07	Uncharacterized protein
Q23614	CELE_ZK822.2	2,09	1,07	4,04E-02	Uncharacterized protein
Q69Z12	mlc-7	2,09	1,06	3,73E-03	Myosin Light Chain
Q93805	ttr-31	2,08	1,05	4,22E-02	TransThyretin-Related family domain
Q9XUW5	ddx-17	2,07	1,05	2,73E-04	DEAD boX helicase homolog
Q965V4	xpo-2	2,05	1,04	3,69E-04	EXPOrtin (Nuclear export receptor)
O01530	asp-6	2,04	1,03	2,38E-06	Aspartic protease 6
Q18823	lam-2	2,03	1,02	2,37E-06	Laminin-like protein lam-2
D1MN68	gpb-1	2,00	1,00	4,35E-02	G Protein, Beta subunit
G5ECR7	elb-1	2,00	1,00	2,04E-02	ELongin B
Q9U2F2	cpt-1	2,00	1,00	1,44E-03	Carnitine Palmitoyl Transferase
Q18599	acer-1	1,99	0,99	1,20E-10	ACEtyl-CoA Regulator
Q20762	F54D1.6	1,99	0,99	3,44E-02	Uncharacterized protein F54D1.6
O76565	F13C5.5	1,97	0,98	8,84E-03	Uncharacterized protein F13C5.5
Q9XVR7	mcm-3	1,97	0,98	6,89E-04	DNA helicase
Q5WRM0;Q9XUT9	CELE_K06G5.1	1,95	0,96	1,08E-03	Uncharacterized protein
P49029	mag-1	1,94	0,96	7,07E-06	Protein mago nashi homolog
A0A0K3AXF6;Q9U2U0	uaf-2	1,94	0,95	2,74E-02	U2AF splicing factor
Q18758	klo-1	1,92	0,94	1,42E-03	KLOtho (Mammalian aging-associated protein) homolog
Q21929	dhs-21	1,92	0,94	2,01E-07	L-xylulose reductase
O44565	lam-1	1,92	0,94	3,09E-05	LAMinin related. See also lmb
O44887	inx-13	1,91	0,94	1,45E-03	Innexin
P19826;Q8MPS2	deb-1	1,91	0,93	4,75E-04	Vinculin;DEnse Body
G5EDQ4;G5EDV5;G5EF89;G5EGA4	cle-1	1,91	0,93	1,15E-02	CLE-1C protein;CLE-1B protein;CoLlagen with Endostatin domain;CoLlagen with Endostatin domain
G5ECQ4	sth-1	1,90	0,92	3,55E-02	Novel protein
Q9NEW6	rsp-3	1,89	0,92	4,00E-03	Probable splicing factor, arginine/serine-rich 3
Q03565	baf-1	1,89	0,92	1,43E-04	Barrier-to-autointegration factor 1
Q20264	acs-11	1,88	0,91	3,44E-06	Fatty Acid CoA Synthetase family

UniProt ID	Gene	Lb23/OP 50 ratio	log2 (ratio)	Qvalue	Protein description
Q18529	C39D10.7	1,88	0,91	3,44E-12	Uncharacterized protein
O45812	CELE_T23G11.7	1,88	0,91	4,35E-02	Uncharacterized protein
P90901;P90901-2; P90901-3;P90901-4	ifa-1	1,87	0,90	3,12E-15	Intermediate filament protein ifa-1;Isoform b, c, d of Intermediate filament protein ifa-1
Q27512;Q7JMT5	nex-2	1,86	0,90	7,85E-04	Annexin
A0A061AD21;A0A061AJI9;C1P640; C1P641;G5EEV6; Q21313	epi-1	1,85	0,89	2,05E-09	Uncharacterized protein;Uncharacterized protein;Uncharacterized protein;Uncharacterized protein;Laminin alpha; Laminin-like protein epi-1
O16887	C29G2.6	1,85	0,89	3,11E-02	Uncharacterized protein C29G2.6
H2L0I2;H2L0I3	lfi-1	1,85	0,88	6,62E-39	Lin-5 (Five) Interacting protein
Q9U3C8;Q9U3C8-2	dcn-1	1,85	0,88	2,30E-02	Defective in cullin neddylation protein 1;Isoform b of Defective in cullin neddylation protein 1
Q23089	xpo-1	1,84	0,88	9,41E-03	EXPOrtin (Nuclear export receptor)
Q9NLD1	hrp-2	1,84	0,88	7,49E-09	HnRNP A1 homolog
Q10020	T28D9.1	1,84	0,88	1,21E-02	Uncharacterized protein T28D9.1
P34685	cap-1	1,84	0,88	1,49E-03	F-actin-capping protein subunit alpha
G5EGU1	egl-30	1,83	0,88	4,29E-03	EGL-30
P52652	T24H10.1	1,83	0,87	1,69E-03	Putative transcription elongation factor S-II
Q22492	hex-1	1,83	0,87	4,68E-08	Beta-hexosaminidase A
O46015;Q7JKM0; Q7YTU0	tep-1	1,83	0,87	4,23E-06	TEP (ThiolEster containing Protein)
Q19853	CELE_F28B4.3	1,82	0,86	5,97E-14	Uncharacterized protein
G5EBK3	erm-1	1,82	0,86	3,09E-05	ERM-1B
Q9BPN5	zig-11	1,81	0,86	6,38E-03	2 (Zwei) IG domain protein
Q18817	ben-1	1,81	0,86	3,52E-04	Tubulin beta chain
Q9XXI9	mcm-2	1,80	0,85	5,21E-03	DNA helicase
P30625;P30625-3	kin-2	1,80	0,85	2,05E-03	cAMP-dependent protein kinase regulatory subunit;Isoform c of cAMP-dependent protein kinase regulatory subunit
Q27371	mup-2	1,80	0,85	9,01E-07	Troponin T
Q9N3G0	CELE_Y53G8AR.6	1,80	0,84	3,92E-03	Uncharacterized protein
P34556	cdk-1	1,79	0,84	8,27E-03	Cyclin-dependent kinase 1
Q22129	CELE_T03G6.3	1,79	0,84	1,82E-02	Uncharacterized protein
P17343	gpb-1	1,78	0,83	1,49E-03	Guanine nucleotide-binding protein subunit beta-1
Q23683	CELE_ZK970.7	1,78	0,83	3,50E-04	Uncharacterized protein
O17915	ran-1	1,78	0,83	9,32E-05	GTP-binding nuclear protein ran-1
G5EEL1;G5EFG1; G5EFL5	alp-1	1,77	0,82	4,73E-04	ALP/Enigma encoding
Q18100;Q4PIU9; Q53U86;Q53U87	sax-7	1,76	0,82	1,29E-05	Sensory AXon guidance;Sensory AXon guidance;SAX-7 SHORTFORM;SAX-7 LONGFORM
G5EGK1	tln-1	1,76	0,82	6,89E-03	Talin
O45569	nep-17	1,76	0,82	7,87E-15	NEPrilysin metallopeptidase family
Q21898	vha-1	1,75	0,81	3,60E-03	V-type proton ATPase 16 kDa proteolipid subunit 1
Q18066;Q18066-2	dim-1	1,75	0,80	1,17E-15	Disorganized muscle protein 1;Isoform b of Disorganized muscle protein 1
I2HAG1;Q09581;Q95ZI4	lec-3	1,74	0,80	9,76E-05	Galectin;32 kDa beta-galactoside-binding lectin lec-3;Galectin
Q18211	ran-3	1,73	0,79	7,25E-03	Regulator of chromosome condensation
Q10033;Q10033-2;Q8I4L0	F15G9.1;F15G9.1; CELE_F15G9.1	1,72	0,78	6,33E-04	Uncharacterized protein F15G9.1;Isoform b of Uncharacterized protein F15G9.1;Uncharacterized protein
Q21763	CELE_R05H5.3	1,72	0,78	2,53E-07	Uncharacterized protein
K8ES75	ctg-1	1,72	0,78	3,44E-02	CRAL/TRIO and GOLD domain containing
P12845	myo-2	1,72	0,78	3,04E-10	Myosin-2
G5ECT6	gpc-2	1,72	0,78	1,73E-02	G Protein, Gamma subunit
P36573	lec-1	1,72	0,78	5,32E-18	32 kDa beta-galactoside-binding lectin
Q9GPA1	CELE_F48E3.4	1,71	0,78	2,23E-08	Uncharacterized protein
O45815	act-5	1,71	0,78	1,63E-11	ACTin
Q23050	clik-1	1,71	0,77	1,02E-11	CaLponIn-liKe proteins
H9G2S0;Q19191	CELE_F08B12.4; F08B12.4	1,71	0,77	4,64E-04	Uncharacterized protein;Uncharacterized protein F08B12.4
G5EDM4;G5EE55;G5EFP7;X5LQ26; X5LVA5	nrfl-1	1,70	0,77	8,76E-06	NHERF (Mammalian Na/H Exchange Regulatory Factor) Like
P49595;Q8MNS3	F42G9.1; CELE_F42G9.1	1,70	0,77	4,23E-02	Probable protein phosphatase 2C F42G9.1;Uncharacterized protein
Q20655	ftt-2	1,70	0,76	1,31E-06	14-3-3-like protein 2
Q21702	CELE_R04B5.5	1,70	0,76	3,42E-02	Uncharacterized protein
G5EE12	cash-1	1,70	0,76	3,94E-02	CKA And Striatin Homolog
A9UJN7	maph-1.2	1,69	0,76	2,81E-03	Microtubule-Associated Protein Homolog
H2KZV8;Q9GP94	mlp-1	1,69	0,76	4,75E-02	MLP/CRP family (Muscle LIM Protein/Cysteine-rich Protein)
Q11176	unc-78	1,69	0,76	1,59E-04	Actin-interacting protein 1
G5EGB1	lec-2	1,68	0,75	5,92E-11	Galectin
H2L0K4;Q8WTM0	CELE_Y45G5AL.1	1,68	0,75	6,57E-03	Uncharacterized protein
G5EGB3	ragc-1	1,68	0,74	2,69E-02	RAs-related GTP binding protein C homolog
G5EFP2	CELE_ZK1321.4	1,67	0,74	2,80E-05	Uncharacterized protein
Q9XTT3	scav-3	1,66	0,73	1,28E-06	SCAVenger receptor (CD36 family) related
P91390	ostd-1	1,66	0,73	3,48E-02	Dolichyl-diphosphooligosaccharide--protein glycosyltransferase subunit 2
O76840;O76840-2	mig-6	1,65	0,72	9,62E-26	Papilin;Isoform a of Papilin
Q17473	ttr-18	1,64	0,72	1,96E-02	TransThyretin-Related family domain
O16214	CELE_F17A9.4	1,64	0,72	1,32E-03	Uncharacterized protein
Q22037;Q22037-4	hrp-1	1,64	0,71	2,43E-05	Heterogeneous nuclear ribonucleoprotein A1;Isoform d of Heterogeneous nuclear ribonucleoprotein A1
Q17348	snr-1	1,64	0,71	1,41E-02	Small nuclear ribonucleoprotein Sm D3
Q9TYW1	vha-19	1,63	0,71	2,63E-03	Vacuolar H ATPase
Q9GYF1	unc-27	1,63	0,71	1,65E-11	Troponin I 2

UniProt ID	Gene	Lb23/OP 50 ratio	log2 (ratio)	Qvalue	Protein description
Q9NA39	ccg-1	1,63	0,71	2,55E-03	Conserved Cysteine/Glycine domain protein
Q20616	skpo-1	1,63	0,70	1,77E-03	Peroxidase skpo-1
Q22866-3	lev-11	1,62	0,70	5,02E-04	Isoform b of Tropomyosin isoforms a/b/d/f
E0AHA7;G5EG85;Q9U9J8;S6FN00	unc-70	1,62	0,70	3,36E-08	Spectrin beta chain
P34650	ZK632.4	1,62	0,70	6,23E-03	Probable mannose-6-phosphate isomerase
P91859;P91859-3	maph-1.1	1,62	0,69	3,90E-05	Microtubule-associated protein homolog maph-1.1;Isoform c of Microtubule-associated protein homolog maph-1.1
G5EDB8	vha-4	1,62	0,69	1,43E-02	VHA-4
Q22752	dyrb-1	1,61	0,69	4,83E-02	Dynein light chain roadblock
Q21633	ubc-18	1,61	0,69	2,10E-04	UBiquitin Conjugating enzyme
G5EET8	pud-1.2	1,60	0,68	2,39E-06	Protein Up-regulated in Daf-2(Gf)
P91910	mec-12	1,60	0,68	1,47E-02	Tubulin alpha-3 chain
O02495	snb-1	1,60	0,68	2,65E-04	Synaptobrevin-1
Q18577	C42D4.1	1,59	0,67	4,35E-03	Uncharacterized protein
Q7Z072	tnt-2	1,59	0,67	1,68E-05	TropoNin T
P34686	cap-2	1,59	0,67	4,36E-03	F-actin-capping protein subunit beta
Q5H9M9	hsp-25	1,58	0,66	2,58E-05	Heat Shock Protein
O44827-2	fgt-1	1,58	0,66	2,04E-03	Isoform a of Facilitated glucose transporter protein 1
Q8MXS9;Q9N3S3;Q9N3S4	rnp-6	1,58	0,66	6,38E-03	RNP (RRM RNA binding domain) containing
A0A1N7SYN7;A0A1N7SYN9;<br>A0A1N7SYP3;A0A1N7SYR7	vab-10	1,57	0,65	9,32E-13	Uncharacterized protein
Q23487;Q7Z145	fubl-2	1,57	0,65	3,41E-02	FUBp (FUBP) Like
H2KYJ2	ttr-59	1,57	0,65	2,90E-04	TransThyretin-Related family domain
Q20626;Q565D1;Q565D2	CELE_F49E2.5	1,57	0,65	1,36E-02	Uncharacterized protein
Q21888	CELE_R102.2	1,57	0,65	3,44E-04	Uncharacterized protein
P34696	hsp-16.1	1,57	0,65	2,86E-02	Heat shock protein Hsp-16.1/Hsp-16.11
Q17886	nasp-1	1,56	0,64	7,40E-03	NASP (Human Nuclear Autoantigenic Sperm Protein) homolog
G5EFE3	CELE_H03A11.2	1,56	0,64	3,36E-05	Uncharacterized protein
Q20222;Q27GU2	lbp-3	1,56	0,64	3,36E-04	Fatty acid-binding protein homolog 3;Lipid Binding Protein
G8XYY6;Q23232	wdr-4	1,56	0,64	8,22E-05	tRNA (guanine-N(7)-)-methyltransferase non-catalytic subunit
Q09422	pgam-5	1,56	0,64	4,74E-02	Serine/threonine-protein phosphatase Pgam5, mitochondrial
P34477	ubc-7	1,56	0,64	4,30E-02	Probable ubiquitin-conjugating enzyme E2 7
Q19289	ifb-1	1,56	0,64	1,13E-13	Intermediate filament protein ifb-1
A0A0K3AUC2	lev-11	1,56	0,64	9,74E-13	Uncharacterized protein
P91502	usp-5	1,56	0,64	2,10E-02	Ubiquitin carboxyl-terminal hydrolase
Q5WRT8;Q5WRT9	npp-21	1,55	0,64	1,31E-02	Nuclear Pore complex Protein
G5ECR0	CELE_T25C12.3	1,55	0,63	2,13E-04	Uncharacterized protein
A0A2C9C2Z1;A0A2C9C3A7;<br>Q9N4M4	anc-1	1,54	0,63	3,08E-50	Uncharacterized protein;Uncharacterized protein;Nuclear anchorage protein 1
H2KZA3;V6CKH6;V6CLQ8;V6CLV0	pqn-22	1,54	0,62	6,12E-03	Prion-like-(Q/N-rich)-domain-bearing protein
Q22918	C37C3.2	1,54	0,62	7,44E-03	Eukaryotic translation initiation factor 5
Q1HB04;Q22317	anp-1	1,54	0,62	4,21E-02	Aminopeptidase
P90961	clic-1	1,53	0,62	7,10E-05	Clathrin light chain
A0A0K3AVF2;O61874	dod-19	1,53	0,62	8,04E-03	Downstream Of DAF-16 (Regulated by DAF-16)
Q20363	sip-1	1,53	0,61	1,04E-07	Stress-induced protein 1
Q18680-4	pyp-1	1,53	0,61	5,08E-11	Isoform d of Inorganic pyrophosphatase 1
Q19826	rpb-8	1,53	0,61	2,58E-02	Probable DNA-directed RNA polymerases I, II, and III subunit RPABC3
G5EGP4	vha-6	1,53	0,61	1,43E-02	V-type proton ATPase subunit a
O01806	ssb-1	1,52	0,61	2,45E-03	Sjogren Syndrome antigen B homolog
P53014	mlc-3	1,52	0,60	2,28E-07	Myosin, essential light chain
Q20774	dnj-13	1,52	0,60	7,70E-04	DNaJ domain (Prokaryotic heat shock protein)
O61880	CELE_F59B1.2	1,51	0,60	5,76E-05	Uncharacterized protein
G8JXY9;Q11073	B0416.5	1,51	0,59	1,43E-02	Uncharacterized protein;Uncharacterized protein B0416.5
O45819	gyg-2	1,51	0,59	1,38E-02	GlYcoGenin like
P34690	tba-2	1,50	0,59	6,15E-03	Tubulin alpha-2 chain
Q9N5G9	CELE_K12H6.6	1,50	0,58	1,02E-03	Uncharacterized protein
Q23451	rad-23	1,50	0,58	3,92E-06	Uncharacterized protein
Q9BL19	rpl-17	0,67	-0,58	4,22E-06	60S ribosomal protein L17
P91128	rpl-13	0,67	-0,58	1,67E-07	60S ribosomal protein L13
Q86NC1;Q9N5U1	pygl-1	0,67	-0,59	2,63E-09	Alpha-1,4 glucan phosphorylase;Alpha-1,4 glucan phosphorylase
Q21966	asp-4	0,67	-0,59	7,64E-13	ASpartyl Protease
O61977	ttr-47	0,67	-0,59	6,15E-03	TransThyretin-Related family domain
Q20034	CELE_F35D11.4	0,66	-0,59	3,75E-02	Uncharacterized protein
G5EEI4	asp-1	0,66	-0,59	2,33E-05	ASpartyl Protease
O45864	CELE_T27E9.2	0,66	-0,59	6,43E-03	Cytochrome b-c1 complex subunit 6
Q93934	CELE_R07H5.8	0,66	-0,60	6,15E-06	Uncharacterized protein
Q19057	acdh-12	0,66	-0,60	1,57E-03	Acyl CoA DeHydrogenase
O45946	rpl-18	0,66	-0,60	1,98E-09	60S ribosomal protein L18
O44906	pck-1	0,66	-0,61	4,22E-11	Phosphoenolypyruvate CarboxyKinase
Q21217	gta-1	0,66	-0,61	5,48E-11	Probable 4-aminobutyrate aminotransferase, mitochondrial
Q7YTR9	C27B7.9	0,65	-0,61	2,55E-03	Uncharacterized protein
O61235	ctl-1	0,65	-0,61	8,86E-06	Catalase-2
O44995	CELE_K12C11.1	0,65	-0,63	1,79E-05	Uncharacterized protein
P50432;P50432-2	mel-32	0,65	-0,63	1,30E-06	Serine hydroxymethyltransferase;Isoform a of Serine hydroxymethyltransferase
Q69Z13;Q86D12;Q9XUS4;Q9XUS5;<br>Q9XUS6	CELE_K08E3.5	0,65	-0,63	2,25E-06	UTP--glucose-1-phosphate uridylyltransferase
O02639	rpl-19	0,64	-0,64	1,82E-09	60S ribosomal protein L19
Q21962;Q86LS6	gldc-1	0,64	-0,64	3,02E-06	Glycine cleavage system P protein
Q09365	ZK1320.3	0,64	-0,64	3,64E-02	Uncharacterized protein ZK1320.3

UniProt ID	Gene	Lb23/OP 50 ratio	log2 (ratio)	Qvalue	Protein description
O17271	heh-1	0,64	-0,64	2,90E-02	Putative protein heh-1
G5EE46	CELE_F54D5.12	0,64	-0,65	1,33E-02	Uncharacterized protein
Q19437	upb-1	0,64	-0,65	2,37E-10	UreidoPropionase Beta
Q966C6;Q966C6-2	rpl-7A	0,63	-0,66	4,83E-06	60S ribosomal protein L7a;Isoform c of 60S ribosomal protein L7a
Q9NAB0	gst-39	0,63	-0,66	2,60E-02	Glutathione S-Transferase
O01578	mpc-2	0,63	-0,66	1,21E-02	Probable mitochondrial pyruvate carrier 2
Q23621	gdh-1	0,63	-0,66	2,00E-24	Glutamate dehydrogenase
P52014	cyn-6	0,63	-0,67	1,61E-02	Peptidyl-prolyl cis-trans isomerase 6
O01812	lbp-6	0,63	-0,67	3,95E-10	Fatty acid-binding protein homolog 6
Q03577	dars-1	0,63	-0,67	5,84E-03	Aspartate--tRNA ligase, cytoplasmic
Q9Y041	hgo-1	0,63	-0,68	5,17E-05	Homogentisate 1,2-dioxygenase
P98080	ucr-1	0,62	-0,68	9,39E-14	Cytochrome b-c1 complex subunit 1, mitochondrial
Q94272	fah-1	0,62	-0,69	4,05E-05	FumarylAcetoacetate Hydrolase
O44512	isp-1	0,62	-0,69	7,58E-05	Cytochrome b-c1 complex subunit Rieske, mitochondrial
Q8MXI1	ucr-11	0,62	-0,69	3,42E-03	Ubiquinol-Cytochrome c oxidoReductase complex
P34662	rpl-35	0,62	-0,70	1,83E-05	60S ribosomal protein L35
O62388	CELE_W01D2.1	0,61	-0,70	1,70E-02	Ribosomal protein L37
Q21355	gst-4	0,61	-0,70	1,89E-06	Glutathione S-transferase 4
Q9XW17	car-1	0,61	-0,70	6,27E-10	Cytokinesis, Apoptosis, RNA-associated
Q23381	mmcm-1	0,61	-0,71	4,58E-03	Probable methylmalonyl-CoA mutase, mitochondrial
H2L0B6;Q21956	CELE_R12C12.9	0,61	-0,71	3,15E-02	Uncharacterized protein
O62146	F09B12.3	0,61	-0,72	1,42E-03	Putative phospholipase B-like 2
Q09450	C05C10.3	0,60	-0,73	2,46E-06	Probable succinyl-CoA:3-ketoacid coenzyme A transferase, mitochondrial
G5EEH6	ivd-1	0,60	-0,74	1,59E-05	Isovaleryl-CoA dehydrogenase
D4YWC3	dct-16	0,60	-0,75	1,85E-12	DAF-16/FOXO Controlled, germline Tumor affecting
B7WNA0	pyk-1	0,58	-0,78	6,26E-14	Pyruvate kinase
O61790	R12E2.11	0,58	-0,78	8,45E-03	Orotate phosphoribosyltransferase
O62277	dct-18	0,58	-0,78	6,18E-04	DAF-16/FOXO Controlled, germline Tumor affecting
O02089	msra-1	0,58	-0,79	2,61E-05	Methionine Sulfoxide Reductase A
O16521	hpo-19	0,58	-0,79	5,02E-06	NADH-cytochrome b5 reductase
H2KZD5;Q27GU1	csr-1	0,57	-0,80	1,26E-03	Chromosome-Segregation and RNAi deficient
G5EGP8	cpz-1	0,57	-0,80	7,83E-04	CathePsin Z
Q9UAW8;W6RTL8	CELE_T12B3.3; CELE_Y40C5A.4	0,57	-0,81	6,65E-03	Uncharacterized protein
O62122	gcsh-1	0,56	-0,82	2,41E-02	Glycine cleavage system H protein
Q22993	pmt-2	0,56	-0,82	7,81E-06	Phosphoethanolamine MethylTransferase
Q18853	cyc-1	0,56	-0,83	7,00E-05	CYtochrome C
O17345	ttr-6	0,56	-0,83	1,24E-04	TransThyretin-Related family domain
Q19478	far-3	0,56	-0,84	1,40E-07	Fatty Acid/Retinol binding protein
Q21770	wago-1	0,55	-0,85	2,80E-04	Argonaute protein wago-1
Q93619	bckd-1b	0,55	-0,85	1,01E-02	Branched Chain Keto acid Dehydrogenase e1 (E1) subunit
Q20502	haly-1	0,55	-0,86	1,63E-02	Histidine ammonia-lyase
P34559	ech-6	0,55	-0,87	8,09E-09	Probable enoyl-CoA hydratase, mitochondrial
Q22100	kat-1	0,55	-0,88	7,63E-08	Acetyl-CoA acetyltransferase homolog, mitochondrial
G5ED31;G5EDD1	ucr-2.1	0,54	-0,88	1,22E-06	Ubiquinol-Cytochrome c oxidoReductase complex
O17680	sams-1	0,54	-0,88	1,27E-17	Probable S-adenosylmethionine synthase 1
G5EET3	cdr-6	0,54	-0,88	1,67E-03	CaDmium Responsive
O44400	F37C4.5	0,54	-0,90	1,30E-31	Protein F37C4.5
P34383	far-2	0,53	-0,92	8,31E-54	Fatty-acid and retinol-binding protein 2
O18000	pes-9	0,52	-0,93	1,97E-10	Patterned Expression Site
O17795;Q17703	stdh-2;stdh-1	0,52	-0,94	1,95E-04	Putative steroid dehydrogenase 2;Putative steroid dehydrogenase 1
Q9XVF7	rpl-2	0,52	-0,94	2,22E-06	60S ribosomal protein L8
P34528	K12H4.7	0,52	-0,96	1,55E-03	Putative serine protease K12H4.7
Q7Z1Q3	alh-12	0,51	-0,96	4,00E-09	ALdehyde deHydrogenase
Q9U2M4	hphd-1	0,51	-0,96	6,46E-19	Hydroxyacid-oxoacid transhydrogenase, mitochondrial
Q21603	ugt-62	0,51	-0,98	1,20E-02	UDP-glucuronosyltransferase
Q19246	dhs-25	0,51	-0,98	5,25E-05	DeHydrogenases, Short chain
Q22633	hpd-1	0,51	-0,98	6,83E-06	4-hydroxyphenylpyruvate dioxygenase
Q19554	abcf-1	0,51	-0,98	4,03E-02	ABC transporter, class F
Q9XXR4	ttr-24	0,50	-1,00	3,21E-04	TransThyretin-Related family domain
E9P851	mctp-1	0,50	-1,01	2,29E-02	Multiple C2 and Transmembrane region Protein family homolog
Q22111	mmaa-1	0,50	-1,01	2,60E-02	Methylmalonic aciduria type A homolog, mitochondrial
Q22814	gst-13	0,50	-1,01	8,22E-03	Glutathione S-Transferase
P52713	alh-8	0,49	-1,02	3,62E-13	Probable methylmalonate-semialdehyde dehydrogenase [acylating], mitochondrial
Q09652	gstk-1	0,49	-1,03	1,82E-02	Glutathione S-transferase kappa 1
A5JYX5-2	dhs-3	0,48	-1,05	1,28E-05	Isoform a of Protein dhs-3
Q23068	CELE_W01A11.1	0,48	-1,06	6,12E-09	Epoxide hydrolase
Q9GUF2	acp-6	0,48	-1,07	2,95E-12	ACid Phosphatase family
G5EDV7	CELE_Y53H1B.2	0,47	-1,08	8,30E-04	Uncharacterized protein
Q19278;Q8MNT7	hach-1	0,46	-1,11	6,44E-11	3-hydroxyisobutyryl-CoA hydrolase, mitochondrial;3-hydroxyisobutyryl-CoA hydrolase, mitochondrial
Q9N5E4	CELE_T02H6.11	0,46	-1,11	8,93E-05	Uncharacterized protein
P34689	glh-1	0,46	-1,12	3,27E-08	ATP-dependent RNA helicase glh-1
Q9GZE9	ldp-1	0,46	-1,12	1,46E-10	Lipid droplet localized protein
Q22968	gcst-1	0,46	-1,13	3,04E-04	Aminomethyltransferase
C1P622;Q09517;S6FCZ1	let-767	0,46	-1,13	2,10E-05	Uncharacterized protein;Very-long-chain 3-oxooacyl-coA reductase let-767;Uncharacterized protein
P91398	cey-3	0,46	-1,13	1,39E-05	C. Elegans Y-box

UniProt ID	Gene	Lb23/OP 50 ratio	log2 (ratio)	Qvalue	Protein description
Q17761	T25B9.9	0,46	-1,14	1,41E-07	6-phosphogluconate dehydrogenase, decarboxylating
Q18938	gst-42	0,45	-1,14	4,99E-04	Probable maleylacetoacetate isomerase
O44503;U4PFH6	aass-1	0,45	-1,16	1,93E-04	AminoAdipate-Semialdehyde Synthase homolog
G5EC10	lec-9	0,44	-1,19	4,70E-10	Galectin
Q86NE0	asp-2	0,44	-1,19	6,19E-06	ASpartyl Protease
O45346	osm-11	0,43	-1,23	9,86E-04	Uncharacterized protein
Q95YF3	cgh-1	0,42	-1,24	3,16E-25	ATP-dependent RNA helicase cgh-1
Q9BL43	CELE_Y71H2AM.13	0,42	-1,25	1,06E-03	Uncharacterized protein
P91306	cey-2	0,42	-1,25	8,81E-13	C. Elegans Y-box
I2HA98;P90925	pah-1	0,42	-1,25	1,62E-06	PhenylAlanine Hydroxylase;Probable phenylalanine-4-hydroxylase 1
Q9NAB4	gst-26	0,42	-1,26	2,05E-02	Glutathione S-Transferase
Q22505	immt-1	0,41	-1,28	4,83E-02	MICOS complex subunit MIC60-1
Q20950	CELE_F57F5.1	0,41	-1,28	4,12E-02	Uncharacterized protein
P53585	acly-1	0,40	-1,32	8,02E-05	Probable ATP-citrate synthase
Q18026	flu-2	0,39	-1,37	3,93E-08	Kynureninase
A0A1I6CM93;Q22133	tag-243	0,37	-1,42	1,34E-02	Uncharacterized protein
P05690	vit-2	0,37	-1,42	0,00E+00	Vitellogenin-2
Q9NAB3	gst-27	0,37	-1,43	2,72E-02	Glutathione S-Transferase
H2KZV5	CELE_T21H3.1	0,36	-1,47	8,79E-04	Uncharacterized protein
O02108;Q336L8	cdc-37	0,36	-1,48	6,83E-03	Probable Hsp90 co-chaperone cdc37;Cell Division Cycle related
H2KZL0;H2KZL1;Q9UA61	CELE_W04B5.3	0,35	-1,50	9,43E-03	Galectin
Q9N384	lec-6	0,35	-1,50	1,26E-13	Galectin
Q18040	oatr-1	0,34	-1,54	1,51E-13	Probable ornithine aminotransferase, mitochondrial
Q19130;Q95QM8	gfat-1	0,34	-1,56	1,56E-03	Glutamine-Fructose 6-phosphate AminoTransferase homolog
Q86FL8	spp-5	0,33	-1,58	5,24E-07	SaPosin-like Protein family
Q19699	gfat-2	0,33	-1,58	1,02E-07	Glutamine-Fructose 6-phosphate AminoTransferase homolog
Q22957	F10G2.1	0,32	-1,66	4,89E-02	UPF0376 protein F10G2.1
P19974	cyc-2.1	0,31	-1,70	3,75E-08	Cytochrome c 2.1
P90889	CELE_F55H12.4	0,30	-1,72	9,93E-08	Uncharacterized protein
P18948;P18948-2	vit-6	0,30	-1,76	0,00E+00	Vitellogenin-6;Isoform a of Vitellogenin-6
Q93315	cytb-5.1	0,29	-1,79	2,93E-03	YTochrome B
P43510;Q8MQC6	cpr-6	0,29	-1,80	7,77E-13	Cathepsin B-like cysteine proteinase 6;Cysteine PRotease related
O02215	cbl-1	0,28	-1,83	1,43E-02	Cystathionine Beta Lyase
P46562	alh-9	0,28	-1,86	2,71E-31	Putative aldehyde dehydrogenase family 7 member A1 homolog
P41877	isw-1	0,27	-1,90	4,65E-02	Chromatin-remodeling complex ATPase chain isw-1
G5ECL3	prp-21	0,26	-1,93	2,93E-03	Pre-RNA processing 21
Q9U3Q6	ugt-22	0,25	-1,99	1,82E-02	UDP-glucuronosyltransferase
P55216	cth-2	0,18	-2,49	1,06E-06	Putative cystathionine gamma-lyase 2
P55155	vit-1	0,18	-2,51	4,77E-12	Vitellogenin-1
O01467	C04E6.5	0,14	-2,84	2,69E-02	Uncharacterized protein
P06125	vit-5	0,12	-3,02	4,42E-67	Vitellogenin-5
O17725	CELE_D1086.3	0,07	-3,79	3,16E-03	Uncharacterized protein
P18947	vit-4	0,07	-3,83	1,63E-316	Vitellogenin-4
Q9N4J2	vit-3	0,06	-4,17	1,08E-04	Vitellogenin-3

Table S4. GO enrichment analysis of proteins regulated between Lb21 and OP50-fed animals. GO terms for biological process were applied for the enrichment and Holm-Bonferroni test correction. The analysis was made using Wormbase WormMine.

GO Term	p-value
**Up in Lb21 vs OP50**	
cellular component organization	0.000001017313318119243
cellular component organization or biogenesis	0.0000017535588143407388
organelle organization	0.0004567495982518751
chromosome organization	0.0007225474986842823
chromatin organization	0.0008999848036831534
chromatin remodeling	0.0018729112205596117
cellular protein-containing complex assembly	0.003400782727311353
protein-containing complex assembly	0.0034274874542153826
RNA splicing	0.006106919181321311
RNA splicing, via transesterification reactions	0.009304666640723882
RNA splicing, via transesterification reactions with bulged adenosine as nucleophile	0.009304666640723882
mRNA splicing, via spliceosome	0.009304666640723882
protein-containing complex subunit organization	0.009678931766197298
DNA conformation change	0.017297380645726525
embryo development ending in birth or egg hatching	0.01848370721419164
cellular component assembly	0.02707681487577866
mRNA processing	0.032631535567242145
protein-DNA complex assembly	0.04068924956611012
multicellular organism development	0.05740695064932772
anatomical structure development	0.10301290759173488
cell cycle	0.1100555911271732
protein-DNA complex subunit organization	0.11239108341645751
mitotic cell cycle	0.14307117703235073
regulation of actin filament depolymerization	0.17632094462446374
cellular component biogenesis	0.2117288290627112
developmental process	0.23915472405451305
mitotic cell cycle process	0.2999436417273276
mRNA metabolic process	0.3577956773298709
pre-replicative complex assembly involved in nuclear cell cycle DNA replication	0.3715463388437246
pre-replicative complex assembly	0.3715463388437246
pre-replicative complex assembly involved in cell cycle DNA replication	0.3715463388437246
cell cycle process	0.40207891841373977
ATP-dependent chromatin remodeling	0.43322625904097367
epithelium development	0.5498707023174321
actin filament organization	0.6537768030233341
negative regulation of organelle organization	0.7731240887947824
regulation of protein depolymerization	0.8913141393606872

**Down in Lb21 vs OP50**	
alpha-amino acid catabolic process	1,73E+08
cellular amino acid catabolic process	3,99E+08
oxidation-reduction process	4,03E+08
alpha-amino acid metabolic process	1,10E+07
small molecule metabolic process	0.000007910564474668729
organic acid catabolic process	0.000008897367885061359
carboxylic acid catabolic process	0.000008897367885061359
organonitrogen compound catabolic process	0.0000093613538022047
carboxylic acid metabolic process	0.000013721590259318608
cellular amino acid metabolic process	0.00001911233453210145
catabolic process	0.00002760882048968361
organic substance catabolic process	0.000056479314184191006
oxoacid metabolic process	0.00006784882802413101
mitochondrial electron transport, ubiquinol to cytochrome c	0.00012099944507635203
small molecule catabolic process	0.00024327555958363585
organic acid metabolic process	0.0002928856607422161
metabolic process	0.0026490345834080083
L-phenylalanine metabolic process	0.0036407070109367437
L-phenylalanine catabolic process	0.0036407070109367437
erythrose 4-phosphate/phosphoenolpyruvate family amino acid metabolic process	0.0036407070109367437
erythrose 4-phosphate/phosphoenolpyruvate family amino acid catabolic process	0.0036407070109367437
organonitrogen compound metabolic process	0.00415817932118776
tyrosine metabolic process	0.010751845958574103
sulfur compound metabolic process	0.016370479664891243
cofactor metabolic process	0.022012043825975162
drug metabolic process	0.025421962867567354
tyrosine catabolic process	0.05605693380765347
valine metabolic process	0.05605693380765347
cellular catabolic process	0.0632360013159324
drug catabolic process	0.11112475873415989
aromatic amino acid family catabolic process	0.14741257488386564
glutathione metabolic process	0.23853629036808935
aromatic amino acid family metabolic process	0.5782813396666809
carboxylic acid biosynthetic process	0.6591549733024149
organic acid biosynthetic process	0.6970734433625344
branched-chain amino acid metabolic process	0.8852662089593908
ATP synthesis coupled electron transport	0.98728015915758
mitochondrial ATP synthesis coupled electron transport	0.98728015915758

Table S5. Primers for *C. elegans* genotyping.

Mutant	Fwd primer sequence, 5'-3'	Rev primer sequence, 5'-3'
daf-16 (mu86)	tccgtgtgcgtttctttctc	ggaaggttggtagaagaacga (wt)<br>cgttcagtgtacgcc (mutant)
pmk-1 (km25)	ccatgacctcagagcctctt	ttggctccgttacctggaat (wt)<br>tgtaccatcttgttgccacg (mutant)
dbl-1 (nk3)	ccttgctgtgtgcctactga (wt)<br>agattaccgctctgccaaat (mutant)	gcgacatagaagcgggtatc
tol-1 (nr2033)	cggttccgctgttgagttat	cagcgttttccactcgttct (wt)<br>tcaaaaatataatcgctggtcaa (mutant)
acs-22 (tm3236)	ggttacaaaatgggcgacgt	gagccgtacaactctccaat
asp-6 (tm2213)	catcacgtattactccggat	caaacggcggactcgttaat
vit-2 (ok3211)	tcacatggaaaacgaggaca (from CGC)	gctcttggttgagaagacgg (from CGC)
clec-65 (ok2337)	agcagcatcgccaactctat	gcaaattgccggaattaaaa
srp-7 (ok1090)	caacataacctttcgtcgca (from CGC)	ccgcaacagctacagtacca (from CGC)

Table S6. TaqMan Assay ID for pig PCR.

**Markers Pig**	**TaqMan Assay ID**
TGF beta-1	Ss04955543_m1
House-keeping	
Beta-Actin	Ss03376563_uH
HPRT	Ss03388274_m1
Ubiquitin	Ss03383372_g1

Supplementary methods

DNA sequencing

Sequencing was performed by the company DNASense.

Illumina library preparation and DNA sequencing

Sequencing libraries were prepared using the NEB Next Ultra II DNA library prep kit for illumina (New England Biolabs, USA) following the manufacturer's protocol. The sequencing libraries were pooled in equimolar concentrations and diluted to 4 nM. The samples were paired end sequenced (2x301bp) on a MiSeq (Illumina, USA) using a MiSeq Reagent kit v3, 600 cycles (Illumina, USA) following the standard guidelines for preparing and loading samples on the MiSeq.

Genome assembly and annotation

The illumina sequence reads were trimmed for adaptors using cutadapt (v. 1.16, (Martin, 2011)). The trimmed reads were assembled using megahit (v. 1.1.3, (Li et al., 2016)). The reads were mapped back to the assembly using minimap2 (v. 2.12-r827 (Li, 2017)) to generate coverage files. Genome visualization was carried out using the mmgenome2 package (v. 2.0.12, (Karst et al., 2016)) in R (v. 3.5.1, (R Core Team, 2018)) using the R-studio environment. Genomes were refined and the final bins were annotated using PROKKA (v. 1.14-dev, (Seemann, 2014)), and completeness was estimated using CheckM (Parks et al., 2015). The genomes were classified using GTDB-TK (v. 0.2.2, (Parks et al., 2018)). Genomes were aligned using fastANI (v. 1.1, (Jain et al., 2018)). Protein orthology was computed using Proteinortho (v. 5.16b, (Lechner et al., 2011)).

Mass spectrometry

Mass spectrometry-based quantification of host-response

A combination of data dependent analysis (DDA) and data independent analysis (DIA) tandem liquid chromatography MS (LC-MS/MS) analysis was applied to identify and quantify proteins. In DDA, precursor ions from the MS scan are selected in a semi-random manner, typically isolating and fragmenting only the most abundant ions for fragmentation and MS/MS analysis, resulting in an incomplete coverage of complex samples. In the DIA-based sequential window acquisition of all theoretical spectra (SWATH) method, practically all precursor ions are fragmented using selected mass ranges instead of single *m/z* ratios for MS filtering. Theoretically, time-resolved MS and MS/MS data is obtained for all precursors in order to support identification and quantification of proteins from even complex tissue samples. However, SWATH analysis results in highly complex MS/MS spectra: hence, for protein identification, SWATH analysis requires a spectral library based on DDA data containing fragmentation patterns for all peptides (Hu et al., 2016). In this study, the

spectral library was based on DDA data from the original sample-set and built prior to the main SWATH analysis.

Replicate analysis (*n*=4 for Lb21 and Lb23 and *n*=3 for OP50) of each of the three sample groups was used to generate a spectral library as well as to make protein identifications and relative quantifications.

Growth and harvest of nematodes for proteome analysis

To prepare samples for proteome analysis, three-days-old *rrf-3* developed on UV-treated OP50 were carefully washed in S-basal and shifted to either Lb21, Lb23 or OP50 seeded NGM plates. On day five, approximately 1000 worms per replicate were washed in S-basal and gently pelleted. Supernatants were aspirated and worm pellets were snap-frozen in liquid nitrogen and stored at -80°C for subsequent protein extraction and proteome analysis. The sample date was selected based on the MRSA killing assay, where worms were also pre-conditioned with Lb21, Lb23 or OP50 for two days prior to pathogen exposure.

Nematode disruption and sample preparation for LC-MS/MS analysis

Frozen worm pellets were thawed on ice and lysed in one pellet-volume of 50 mM Triethylammonium bicarbonate (Sigma-Aldrich/Merck, Missouri, United States) + 1% Sodium deoxycholate (Sigma-Aldrich/Merck), pH 8.5. 0.3 g of different-sized ceramic/glass beads (B. Braun Biotech International AG, Melsungen, Germany) were added and samples were homogenized by beat-beating using a Precellys 24 tissue homogenizer (Bertin Instruments, Montigny-le-Bretonneux, France) in three cycles of 3x 6,000 rpm for 20 sec, with 30 sec breaks in between runs. Samples were snap-frozen in liquid nitrogen in between the three cycles to avoid sample warm-up and to increase cell lysis. Samples incubated 10 min at 95° C to increase protein extraction and subsequently cooled on ice. Homogenates were centrifuged at 13,000 rpm for 20 min, 4° C to pellet worm and cell debris, and supernatants were collected in LoBind tubes (Eppendorf AG, Hamburg, Germany). Protein concentrations were measured using the Pierce BCA protein assay kit (Thermo Fisher Scientific, MA, Unites States) with bovine serum albumin as standard. Cysteine residues were reduced in 10 mM dithiothreitol for 30 min at 56°C, followed by alkylation of the formed thiol groups in 55 mM iodoacetamide for 30 min in the dark at 20°C. Protein concentration were adjusted to 1 µg/µL, and enzymatic digestion of the proteins was performed by the addition of trypsin (Sigma-Aldrich/Merck) (enzyme to protein ratio of 1:50 w/w) followed by over-night digestion at 37°C. Sodium deoxycholate was subsequently removed by acid precipitation with formic acid (pH<2.5), samples were centrifuged at 13,000 rpm for 20 min at 4° C and soluble peptides were recovered.

LC-MS/MS analysis

DDA and SWATH runs were performed in a concatenated fashion for all samples. Synthetic indexed retention time (iRT) peptide standards (Biognosys, Schlieren, Switzerland) were spiked into all digested samples in a 1:25 ratio (v/v) to support accurate retention time calibration between individual files. For building the spectral library, LC-MS/MS analyses of all replicates were performed using the DDA method. For each sample, 5 µg peptide material was injected into an Eksigent NanoLC 415 system (AB/Sciex, MA, United States) coupled to TripleTOF 6600 mass spectrometer (AB/Sciex) and a DuoSpray Ion Source (AB/Sciex) controlled by Analyst TF 1.71 software (AB/Sciex). The Eksigent NanoLC 415 system controls the gradients of the buffers and contains a YMC-Triart C18 trap column (0.5 mm x 5.0 mm, 3 µm particles, YMC) and a C18 analytical column (0.3 mm x 150 mm, 3 µm particles, YMC). The trap column was used to purify the peptides before eluting them onto the analytical column and the analytical column to separate the peptides prior to the MS analysis. An 87 min gradient from 95% to 20% buffer A (0.1% formic acid in water) and from 5% to 80% buffer B (0.1% formic acid in acetonitrile) at a flow rate of 5 µL/min was applied for chromatography. The DDA mode was applied over a mass range of 400-1250 *m/z* with an accumulation time of 0.25 s, followed by product ion scans of a maximum of 30 candidate ions within each cycle. The following settings were used: Floating ion spray voltage of 5500 V, curtain gas of 25 psi, ion source gas 1 of 10 psi, collision energy of 10 V, and declustering potential of 90 V.

For the SWATH analysis, 5 µg peptide material was analyzed using the same technical set up as for the DDA analyses with identical chromatographic parameters, but with a gradient of 57 min. SWATH-MS analysis was performed in a looped product ion mode, with a set of 100 consecutive windows across a range of *m/z* values from 400-1250. Accumulation time was 0.25 s for the TOF-MS scan and 25 ms for the product ion scan.

Spectral library generation and data analysis

A spectral library was created based on DDA files from all sample groups. Unprocessed, raw *.wiff* files were searched as a merged file using ProteinPilot 5.0.1 (AB/Sciex) against a search database containing sequences from *C. elegans* (ref. proteome 1940), *E. coli* K12 (ref. proteome 0625), *Levilactobacillus brevis* ATCC 367/JCM (ref. proteome 1170), Ref. proteomes were downloaded from http://www.uniprot.org/ July 31, 2018; 34,862 entries in total. iRT peptide and general contaminants sequences were added to the database.

Search parameters were as follow: Fixed modifications = alkylation of cysteines (iodoacetamide); digestion = trypsin; instrument = TripleTOF 6600; search effort = thorough ID; confidence scores > 0.05, which equals 1% FDR at the peptide level. Protein detections were accepted when the ProtScore (given by the Protein Pilot software) was >1.3 (equivalent to 95% confidence) and a

minimum of two peptides per protein was set as criteria for confident protein assignment.
The ProteinPilot-generated *.group* file was imported as a spectral library into Spectronaut Pulsar (Biognosys). Spectronaut is a proteomics software aimed at analyzing DIA proteomics experiments and was used to identify and make relative quantifications (and comparisons between sample groups) of proteins based on the MS-SWATH data and the DIA-based spectral library. Spectronaut default settings were applied, and for identification, protein and precursor Qvalue cut offs were set to 0.01, corresponding to an FDR of 1%, and Qvalue percentile for quantification was set to 0.7 (full precursor profile across 70% of all samples). Differential abundance grouping was calculated based on precursor ions (summed fragment ions) and t-tests were performed on log(2) ratios of these quantities, and proteins regulated between Lb21/Lb23/OP50 (Qvalue<0.05 and log2 ratio > 0.58 or log2 ratio < -0.58) were returned by Spectronaut. A heat map was generated using the free software Heatmapper and based on Euclidean Distance Measurement Method and the Average Linkage Clustering Method. To visualize the grouping of the regulated protein, the free software Venny was applied. Proteins were grouped according to regulation, and functionally annotated using the Gene Ontology (GO) terminology and enrichment analyses of all groups (up- and down regulated in Lb21 versus Lb23 and Lb21 versus OP50) were performed using Wormbase WS273 Intermine data mining platform for *C. elegans* and related nematodes. For enrichment analysis, a Holm-Bonferroni test correction was applied and only GO terms for biological process.

References:

Jain, C., Rodriguez-R, L.M., Phillippy, A.M., Konstantinidis, K.T., and Aluru, S. (2018) High throughput ani analysis of 90K prokaryotic genomes reveals clear species boundaries. *Nature communications* **9**: 5114.

Karst, S.M., Kirkegaard, R.H., and Albertsen, M. (2016) Mmgenome: a Toolbox for Reproducible Genome Extraction From Metagenomes. *bioRxiv* 059121.

Lechner, M., Findeiß, S., Steiner, L., Marz, M., Stadler, P.F., and Prohaska, S.J. (2011) Proteinortho: Detection of (co-) orthologs in large-scale analysis. *BMC bioinformatics* **12**: 124.

Li, D., Luo, R., Liu, C.-M., Leung, C.-M., Ting, H.-F., Sadakane, K., et al. (2016) MEGAHIT v1. 0: A fast and scalable metagenome assembler driven by advanced methodologies and community practices. *Methods* **102**: 3–11.

Li, H. (2017) Minimap2: Fast pairwise alignment for long dna sequences. *arXiv preprint arXiv:1708.01492*.

Martin, M. (2011) Cutadapt removes adapter sequences from high-throughput sequencing reads. *EMBnet.journal* **17**: 10.

Parks, D.H., Chuvochina, M., Waite, D.W., Rinke, C., Skarshewski, A., Chaumeil, P.-A., and Hugenholtz, P. (2018) A standardized bacterial taxonomy based on genome phylogeny substantially revises the tree of life. *Nature biotechnology*.

Parks, D.H., Imelfort, M., Skennerton, C.T., Hugenholtz, P., and Tyson, G.W. (2015) CheckM: Assessing the quality of microbial genomes recovered from isolates, single cells, and metagenomes. *Genome research* gr–186072.

R Core Team (2018) R: A language and environment for statistical computing R Foundation for Statistical Computing, Vienna, Austria.

Seemann, T. (2014) Prokka: Rapid prokaryotic genome annotation. *Bioinformatics* **30**: 2068–2069.

Hu, A., Noble, W. S. & Wolf-Yadlin, A. Technical advances in proteomics: New developments in data-independent acquisition. F1000Research (2016) doi:10.12688/f1000research.7042.1.
